# Expression of *Brassica napus TTG2*, a regulator of trichome development, increases plant sensitivity to salt stress by suppressing the expression of auxin biosynthesis genes

**DOI:** 10.1093/jxb/erv287

**Published:** 2015-06-12

**Authors:** Qingyuan Li, Mei Yin, Yongpeng Li, Chuchuan Fan, Qingyong Yang, Jian Wu, Chunyu Zhang, Hong Wang, Yongming Zhou

**Affiliations:** ^1^National Key Laboratory of Crop Genetic Improvement, Huazhong Agricultural University, Wuhan 430070, China; ^2^Department of Biochemistry, University of Saskatchewan, Saskatoon SK S7N 5A2, Canada

**Keywords:** *Arabidopsis*, auxin biosynthesis, *Bna.TTG2*, *Brassica napus*, salt stress, transcription factor, trichome.

## Abstract

WRKY transcription factors (TFs) are plant specific and play important roles in regulating diverse biological processes. To identify TFs with broad-spectrum effects on various stress responses in *Brassica napus*, an important oil crop grown across diverse ecological regions worldwide, we functionally characterized *Bna.TTG2* genes, which are homologous to the *Arabidopsis AtTTG2* (*WRKY44*) gene. Four *Bna.TTG2* genes were capable of rescuing the trichome phenotypes of *Arabidopsis ttg2* mutants. Overexpressing one *Bna.TTG2* family member, *BnaA.TTG2.a.1*, remarkably increased trichome numbers in *Arabidopsis* and *B. napus* plants. Interestingly, the *BnaA.TTG2.a.1*-overexpressing plants of both species exhibited increased sensitivity to salt stress. In *BnaA.TTG2.a.1*-overexpressing *Arabidopsis* under salt stress, the endogenous indole-3-acetic acid (IAA) content was reduced, and the expression of two auxin biosynthesis genes, *TRYPTOPHAN BIOSYNTHESIS 5* (*TRP5*) and *YUCCA2* (*YUC2*), was downregulated. The results from yeast one-hybrid, electrophoretic mobility shift, and dual-luciferase reporter assays revealed that BnaA.TTG2.a.1 is able to bind to the promoters of *TRP5* and *YUC2*. These data indicated that BnaA.TTG2.a.1 confers salt sensitivity to overexpressing plants by suppressing the expression of IAA synthesis genes and thus lowering IAA levels. Transgenic *Arabidopsis* plants with an N-terminus-deleted BnaA.TTG2.a.1 no longer showed hypersensitivity to salt stress, suggesting that the N terminus of BnaA.TTG2.a.1 plays a critical role in salt stress responses. Therefore, in addition to its classical function in trichome development, our study reveals a novel role for *Bna.TTG2* genes in salt stress responses.

## Highlight

This study revealed that the *Brassica napus* WRKY transcription factor *Bna.TTG2* participates in both trichome development and auxin-mediated salt stress responses.

## Introduction

Transcription factors (TFs) regulate gene expression by binding to specific *cis*-elements (Latchman, 1997). The WRKY TF family, named after the conserved WRKY domain, is plant specific and includes more than 70 members in *Arabidopsis* (Eulgem *et al.*, 2000). The DNA-binding domain of WRKY TFs binds specifically to the W-box (TTGACC/T) in the promoters of target genes, thereby regulating their transcription (Rushton *et al.*, 2010). The majority of WRKY TFs characterized to date are involved in stress responses (Rushton *et al.*, 2010), while a few members of the family are found to function in plant growth and development. *AtWRKY44* (*AtTTG2*) was the first WRKY TF family member identified as regulating trichome development and proanthocyanidin accumulation in the seed coat (Johnson *et al.*, 2002; Ishida *et al.*, 2007). Another example is *AtWRKY10* (*MINI3*), which is involved in endosperm cellularization and seed development (Luo *et al.*, 2005). It remains unknown whether these WRKY TFs that function in plant development also participate in stress responses.

Soil salinization is a major abiotic stress in modern agriculture, and plants have developed intricate mechanisms at multiple levels to adapt to such stress, including transcriptional regulation upon the stimulus of salt stress (Chen *et al.*, 2012). It has been shown that WRKY TFs play important roles in salt stress responses in plants. For example, the expression of *AtWRKY25* and *AtWRKY33* is induced by salt, and *AtWRKY25* or *AtWRKY33* overexpression in *Arabidopsis* can increase salt tolerance (Jiang and Deyholos, 2009). Similarly, overexpression of *GmWRKY54*, a salt-inducible *WRKY* gene from soybean, results in enhanced tolerance to salt stress in *Arabidopsis* (Zhou *et al.*, 2008). Expression of the rice *OsWRKY45* gene is markedly induced by salt stress, and *35S:OsWRKY45* transgenic *Arabidopsis* plants are more tolerant to salt (Qiu and Yu, 2009).

Among the adaptive mechanisms to salt stress in plants, auxin has been shown to be involved in these responses. Expression profiling of NaCl-stressed *Arabidopsis* roots revealed that auxin biosynthesis-, transport-, and response-related genes are involved in the response to salt stress (Jiang and Deyholos, 2006). Furthermore, it has been suggested that auxin redistribution in *Arabidopsis* modulates root development under salt stress (Wang *et al.*, 2009), and *SALT OVERLY SENSITIVE3* (*SOS3*), a calcium-binding protein gene required for plant salt tolerance, affects lateral root development under low salt stress by regulating auxin redistribution (Zhao *et al.*, 2011). More specifically, endogenous indole-3-acetic acid (IAA) levels in tomato and maize are decreased under salt stress, and such a decline is more dramatic in salt-sensitive genotypes (Dunlap and Binzel, 1996; Zorb *et al.*, 2013). Exogenous IAA or enhanced endogenous IAA production in sorghum and mung bean were found to alleviate growth inhibition when the plants were subjected to salt stress (Azooz *et al.*, 2004; Zahir *et al.*, 2010). However, the role of auxin in the salt stress responses mediated by WRKY TFs has not yet been explored.

*Brassica napus* (AACC, 2*n*=38) is an important oil crop grown across diverse ecological areas worldwide, and its productivity is severely affected by a number of biotic and abiotic stresses, including salinity, pathogens, and insects. Therefore, a long-term goal of *Brassica* genetic improvement involves the identification of genes with broad-spectrum effects on various stress responses. An initial study showed that the expression of WRKY TFs genes in *B. napus* is induced by fungal pathogens and hormone treatments (Yang *et al.*, 2009). Overexpression of *BnWRKY33* in *B. napus* resulted in enhanced resistance to *Sclerotinia sclerotiorum* (Wang *et al.*, 2014). The involvement of *AtTTG2* in trichome development (Johnson *et al.*, 2002) indicates a potential for improving plant tolerance to certain insects by increasing their trichome numbers (Plett *et al.*, 2010), although functional analyses of WRKY TFs in *B. napus* are still rare. Here, we report the molecular characterization of *Bna.WRKY44* (*Bna.TTG2*) genes and demonstrate that these genes participate in trichome development in *B. napus* leaves. We showed that overexpression of *BnaA.TTG2.a.1* caused hypersensitivity to salt stress in both *Arabidopsis* and *B. napus*, resulting in reduced IAA content by suppressing *TRYPTOPHAN BIOSYNTHESIS 5* (*TRP5*) and *YUCCA2* (*YUC2*) expression under salt stress. Our data thus reveal a novel role for *TTG2* genes in the response to salt stress.

## Materials and methods

### Plant materials, growth conditions, and treatments

*B. napus*, *Brassica rapa* and *Brassica oleracea* plants were grown in an isolated nursery field of the Huazhong Agriculture University experimental farm, Wuhan, China. *Arabidopsis* plants, including both Columbia (Col) and Landsberg (Ler) ecotypes, were grown in growth chambers under long-day conditions (16h light/8h dark) under white fluorescent light at 20 °C during the day and 18 °C at night, with a relative humidity of 60%.

The *Arabidopsis* seeds were germinated on agar plates containing half-strength Murashige and Skoog medium (½ MS), 1% (w/v) sucrose, and 0.7% (w/v) agar at pH 5.7. After stratification at 4 °C for 2 d, the seeds were placed on the medium in Petri plates and allowed to grow in an illuminated growth chamber at 23 °C. After 4 d, the seedlings were transferred to ½ MS with different supplementation of NaCl, mannitol, abscisic acid (ABA), or IAA in square plates for stress treatments. For *B. napus*, seeds were germinated on filter paper using ¼ Hoagland nutrient solution (HS; Hoagland and Arnon, 1950) for 5 d and then transferred to ½ HS with a cystosepiment supporter for another 7 d, and finally to HS or HS-NaCl. The HS medium was refreshed every 3 d.

### Gene cloning, sequence alignment, and phylogenetic analysis

Total RNA was extracted from the leaves of J6104 or *Arabidopsis* (Col-0) seedlings using a Plant Total RNA Extraction kit (Biotake). For each sample, 2 μg of total RNA was used for reverse transcription with TransScript First-Strand cDNA Synthesis Super Mix (TransGen). We follow the nomenclature rules of Ostergaard and King (2008) for naming the *Brassica* genes identified in this study.

Sequences of *BraA.TTG2* and *BolC.TTG2* were obtained by searching a *Brassica* database containing *B. rapa* and *B. oleracea* genome sequences (Cheng *et al.*, 2011). Sequence alignment and phylogenetic analyses were performed as described previously (Yang *et al.*, 2012).

### Plasmid constructs and plant transformation

The plasmid constructs for this study were prepared using standard molecular biology techniques, and the details are provided in Supplementary Methods, available at *JXB* online. The constructs were introduced into *Agrobacterium tumefaciens* GV3101 by electroporation. *Arabidopsis* plants were transformed by the floral dipping method (Clough and Bent, 1998). Seeds were harvested and screened on 0.8% agar plates containing ½ MS and 50mg l^–1^ of kanamycin or 25mg l^–1^ of hygromycin. *B. napus* (cultivar J572) plants were transformed as described previously (Zhou *et al.*, 2002). Putative transformants (T0) were transferred to soil. DNA was isolated from young leaves and used to determine the presence of the transgene by PCR.

### β-Glucuronidase (GUS) staining assay

Histochemical staining of GUS activity was performed following a described protocol (Ishida *et al.*, 2007). Seedling samples were incubated in staining solution at 37 °C overnight in the dark and then washed with 9:1 (v/v) ethanol:acetic acid solution before observation, while root samples were incubated for 1h at 37 °C in GUS staining buffer and observed as a whole mount in water. Photographs were taken using a Nikon D40 camera or a Nikon Eclipse80i microscope equipped with a Nicon-DS-Ri1 CCD camera.

### Subcellular localization

To determine the subcellular localization of full-length Bna.TTG2 proteins, *35S:Bna.TTG2s:GFP* constructs [fused to green fluorescent protein (GFP)] were introduced into wild-type (WT) *Arabidopsis* (Col-0). Root tips of 1-week-old T3 homozygous plants were examined under a Nikon Eclipse80i fluorescence microscope first and then imaged under an LSM 510 META confocal microscope (Zeiss).

### Assessment of trichome phenotypes

The first six true leaves of *Arabidopsis* from soil-grown 30-d-old *Arabidopsis* plants were used for determining trichome numbers. The leaves were fixed and cleared of chlorophyll with 70% ethanol, and the epidermal cells were photographed with a Nikon Eclipse80i microscope. The trichome number, cell number, and leaf areas were determined as described previously (Cheng *et al.*, 2013). The trichome index was calculated using the following formula: number of trichomes/(number of trichomes+number of pavement and guard cells)×100 (Maes *et al.*, 2008).

For trichome density (number of trichomes cm^–2^) assessment of *B. napus* leaves, leaf disks of 10.0mm in diameter punched from similar locations of the third and fourth true leaves of WT and transgenic *B. napus* were used, and the number of trichomes was used to calculate the trichome density on each leaf (Gruber *et al.*, 2006).

### Cytohistological analysis

For cellular structure analysis, *Arabidopsis* leaves were fixed in a solution containing 70% ethanol, 5% glacial acetic acid, and 3.7% formaldehyde for 24h at room temperature; this solution was then replaced twice with 70% ethanol. After dehydration through an ethanol series of 80, 90, and 100%, the fixed leaves were embedded into Technovit 7100 resin (Heraeus Kulzer) and polymerized at 37 °C for 3 d. The sample blocks were sectioned into 2 μm thick slices with a microtome (Leica) and stained with 0.25% toluidine blue O (Merck). Images were captured with a Nikon D40 camera or Nikon Eclipse80i microscope equipped with a Nikon DS-Ri1 CCD camera.

### IAA determination

Sample preparation and IAA content quantitation were performed as described previously (Liu *et al.*, 2012; Novak *et al.*, 2012). One-week-old *Arabidopsis* seedlings were harvested, and samples of ~60mg fresh weight were frozen with liquid nitrogen and stored at –80 °C before analysis. The tissues were ground in 800 μl of pre-cooled extraction buffer (methanol:ddH_2_O:acetic acid, 80:19:1, v/v/v) supplied with 1ng indole-3-acetic-2,2-d2 acid (D_2_-IAA; Sigma-Aldrich) as internal standard with ceramic beads (3mm) using a Tissue Lyser (JXFSTPRP-192; China) for 90 s; the mixture was incubated at 4 °C for 12h with shaking in the dark. After centrifugation (10 000*g*) at 4 °C for 10min, the supernatant was transferred to a fresh tube; the pellet was mixed with another 400 μl of pre-cooled extraction buffer without D_2_-IAA, shaken for 2h at 4 °C in the dark, and centrifuged. The combined supernatants were then evaporated with nitrogen gas using a Termovap sample concentrator (N-EVAP 112, Organomation Associates, USA) at room temperature. The pellet was resuspended in 300 μl of pre-cooled 10% methanol and filtered using a syringe-facilitated 13mm diameter nylon filter with a pore size 0.22 μm. Ten microlitres of prepared sample was injected into a Waters Atlantis T3 column (2.1×150cm, 3 μm), separated using a high-performance liquid chromatography system (Shimadzu Corporation) at ambient temperature, and eluted at a constant flow rate of 0.25ml min^–1^ with a binary solvent system consisting of 0.02% acetic acid in H_2_O (solvent A) and 0.02% acetic acid in acetonitrile (solvent B). The gradient series was 0% B for 5min, 0–16% B for 5min, 16–100% B from 8–20min, a hold at 100% B for 5min, 100–0% B for 3min, and 0% B for another 4min for re-equilibration. Detection was performed with a hybrid triple quadrupole/linear ion trap mass spectrometer with an electrospray ion source. The negative ionization mode (ionspray voltage: 4.5kV, source temperature: 550 °C, curtain gas: 35 Psi, collision gas: 8 Psi, ion source gas 1: 30 Psi, ion source gas 2: 60 Psi) was used to scan the product ion fragments of IAA (Liu *et al.*, 2012).

### Transactivation assays in yeast cells

Full-length *BnaA.TTG2.a.1* or *BnAP2* (positive control) cDNA was fused in frame with the yeast GAL4 DNA-binding domain (GAL4BD) in the backbone of pDBLeu (Invitrogen). The construct was introduced in the yeast strain MaV203 (Invitrogen). Positive clones were transferred to a SC–Leu–His plate containing 40 μg ml^–1^ of X-α-gal for colour development.

### Yeast one-hybrid (YIH) analysis

A Y1H assay was performed according to the manual of Matchmaker Gold Yeast One-Hybrid Library Screening System (Clontech). For preparation of bait vectors, the following sequences were fused to pAbAi: a classical W-box (5′-CGTTGACCTTGACCTTGAC TTCGTTGACCTTGACCTTGACTT-3′); a mutated W-box (5′-CGTTGAACTTGAACTTGAATTCGTTGAACTTGAACT TGAATT-3′; mutated nucleotides underlined); a 1193bp promoter sequence upstream of the *TRP5* start codon and its mutated version *mTRP5* for the W-box motifs; and 2003bp of *YUC2* and its mutated version *mYUC2*. For preparation of the prey vector, *BnaA.TTG2.a.1* cDNA was cloned in frame into pGADT7 to yield the BnaA.TTG2.a.1:GADT7 fusion protein. The respective *Bbs*I-cut bait vectors and *BstB*I-cut *P53-AbAi* (positive control) vector were transformed into the Y1HGold yeast strain. After the selection of transformants on SD–Ura plates and determination of the minimal inhibitory concentration of aureobasidin A (AbA; Clontech) for the bait strains, *BnaA.TTG2.a.1:GADT7AD* was introduced into the Y1HGold strain. The co-transformed yeast cells were cultured on SD–Leu plates with or without AbA and incubated at 30 °C until colonies in the positive control (p53) were visible.

### Electrophoretic mobility shift assays

The *BnaA.TTG2.a.1* cDNA was inserted into a pET-28a inducible expression vector (with His tag) and expressed in the *Escherichia coli* BL21 strain. The His–BnaA.TTG2.a.1 recombinant fusion protein was purified using HisPur Ni-NTA Spin Columns (Thermo Scientific). Synthesized oligonucleotides (48–59bp) were labelled using a DIG Gel Shift kit (Roche). DNA–protein binding reactions were performed by incubating 100ng of purified BnaA.TTG2.a.1 recombinant protein with digoxigenin-labelled promoter fragments, as described in the Roche protocol manual. The DNA–protein mixture was incubated at room temperature for 30min and separated on a 12% polyacrylamide gel in Tris/glycine buffer (25mM Tris, 2mM EDTA, 380mM glycine). The hybridizations were detected using a C-Digit Blot Scanner (LI-COR Biosciences).

### Analyses of transcriptional activation/repression and DNA binding in mesophyll protoplasts

The dual-luciferase reporter (DLR) transient expression system was provided by Professor Shouyi Chen (Institute of Genetics and Developmental Biology, Chinese Academy of Sciences, Beijing, China) and DLR assays were performed essentially as described by Hao *et al.* (2011).

To determine whether potential transcriptional activation/repression domains occur in BnaA.TTG2.a.1, the full-length *BnaA.TTG2.a.1* cDNA and a series of deletion constructs were fused with the GAL4BD-coding sequence as effectors. Luciferase (LUC) driven by the 35S promoter was used as a reporter. The *Renilla LUC* gene driven by the *Arabidopsis UBIQUITIN3* (*AtUBI3*) promoter was used as an internal control.

To determine the binding of BnaA.TTG2.a.1 to the promoters of *TRP5* and *YUC2*, the *35S:TTG2:SRDX* construct was used as an effector, *Pro*_*TRP5*_ *:LUC*, *Pro*_*YUC5*_ *:LUC*, *Pro*_*mTRP5*_ *:LUC*, and *Pro*_*mYUC2*_ *:LUC* as reporters, and the *Renilla LUC* gene driven by the *AtUBI3* promoter as an internal control. The *Arabidopsis* mesophyll protoplasts preparation and subsequent transfection were performed according to previously reported protocols (Yoo *et al.*, 2007). After transfection, firefly LUC and *Renilla* LUC were measured using the DLR assay system (Promega).

### Quantitative real-time PCR (qRT-PCR)

*Arabidopsis* seeds were germinated and grown on ½ MS for 4 d and then transferred to ½ MS medium with or without (control) NaCl for 48h. Total RNA was isolated from the treated seedlings with TRIZOL reagent (Invitrogen). For each sample, 5 μg of RNA was treated with 10U of DNase I (Thermo) to remove residual DNA and then used for reverse transcription with TransScript First-Strand cDNA Synthesis Super Mix (TransGen). qRT-PCR was performed as described previously (Li *et al.*, 2012). *Arabidopsis AtACT7* was used as the internal control, and the relative gene expression levels were calculated using the 2^–Δ*C*^_T_ method [–Δ*C*_T_ indicates –[*C*_T of target_ – *C*_T of AtACT7_], *C*_T_ is the threshold cycle number of the amplified gene].

### Statistics

Each graphical plot represents the results of multiple independent experiments (*n*≥3), and the values are means±SE. Statistical significance was determined using two-tailed unpaired Student’s *t*-tests, and *P* values pf <0.05 were considered statistically significant.

### Genomic DNA isolation, Southern blot analysis, and ion content determination

These methods are described in Supplementary Methods.

### Primers

The primers used in this study are listed in Supplementary Table S1, available at *JXB* online.

### GenBank accession numbers

The sequence data from this article can be found in GenBank under accession numbers KJ596447, KJ596448, KJ596449, and KJ596450.

## Results

### Molecular cloning and characterization of *TTG2* genes in *B. napus*

Four *TTG2* homologues were cloned from *B. napus* genomic DNA and cDNA and named *BnaA.TTG2.a.1*, *BnaA.TTG2.b.1*, *BnaC.TTG2.a.1*, and *BnaC.TTG2.b.1.* The number of homologous copies of *TTG2* in *B. napus* was further confirmed by Southern blot analysis (Supplementary Fig. S1, available at *JXB* online). The deduced protein sequences showed high degrees of similarity with *Arabidopsis* TTG2 (Supplementary Fig. S2A, available at *JXB* online). The TTG2 proteins from *B. napus* and its two progenitor diploid species were grouped into two clusters based on their sequences (TTG2.a and TTG2.b; Supplementary Fig. S2B).

**Fig. 1.**
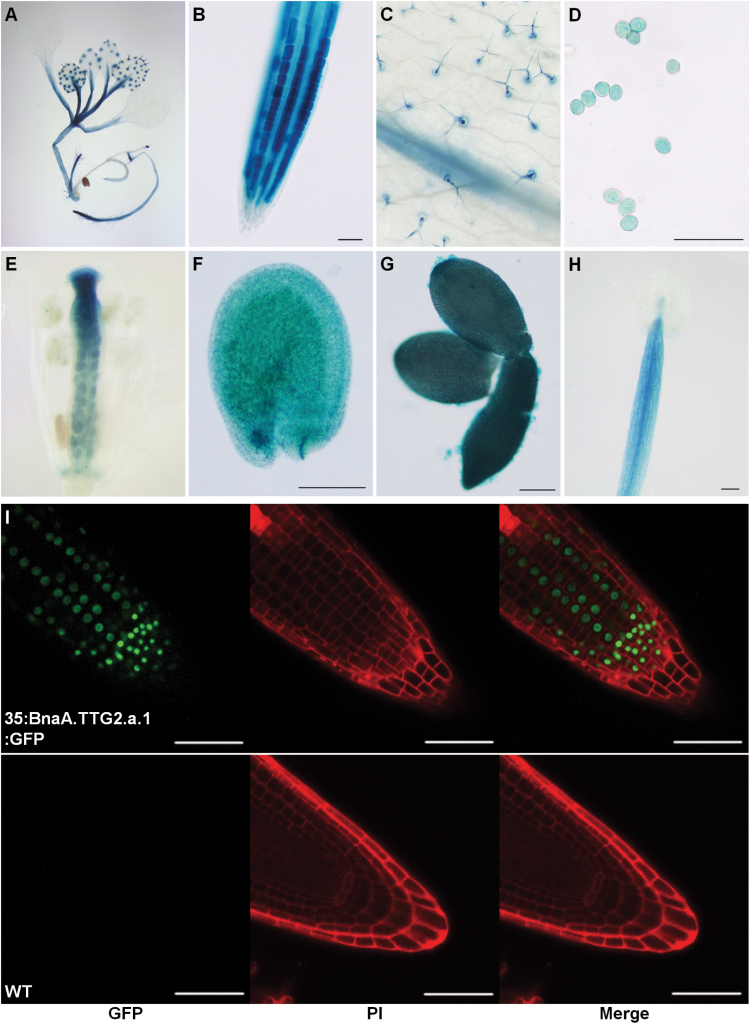
Expression pattern of *Bna.TTG2* genes and subcellular localization of Bna.TTG2.a.1 proteins. (A–G) *BnaA.TTG2.a.1* expression in various tissues. Transgenic plants (15-d-old seedlings) harbouring *Pro*_*BnaA.TTG2.a.1*_ *:GUS* were stained for GUS activity in seedlings (A), roots (B), trichomes (C), pollen (D), developing seeds (E, F), and mature embryos (G). (H) *Pro*_*BnaC.TTG2.a.1*_ *:GUS* expression in the filament. (I) Subcellular localization of the Bna.TTG2:GFP fusion protein. The WT root was used as a control. The GFP images are shown in the left column, propidium iodide (PI) images in the middle, and merged images on the right. Bars, 100 μm (B, D, F, G, and H); 50 μm in (I).

To understand the expression patterns of *Bna.TTG2* genes, four promoter fragments (1707, 1617, 1932, and 1631bp) upstream of the ATG start codon of the four *B. napus TTG2* genes were isolated. Promoter–GUS reporter constructs were used to transform WT *Arabidopsis*, and T3 homozygous lines were used for expression analyses. GUS staining showed that all four *Bna.TTG2* genes were highly expressed in root non-hair cells, root hairs, trichomes, pollen, developing seeds, and mature embryos (Fig. 1A–G). In addition, *BnaC.TTG2.a.1* was also expressed in filaments (Fig. 1H).

To investigate the subcellular localization of the Bna.TTG2 proteins, a *35S:BnaA.TTG2.a.1:GFP* construct was introduced in WT *Arabidopsis*. Confocal laser-scanning microscopy revealed that the BnaA.TTG2.a.1:GFP fusion protein was localized to the nuclei of root cells (Fig. 1I), which is consistent with a previous study on AtTTG2 (Ishida *et al.*, 2007). Similarly, the other three Bna.TTG2:GFP fusion proteins were also found to be localized to the nucleus (Supplementary Fig. S3, available at *JXB* online).

### Overexpression of *BnaA.TTG2* increases trichome numbers in *Arabidopsis* and *B. napus*

To determine whether the *Brassica TTG2* genes have similar functions in trichome development, we used a series of *Bna.TTG2:GFP* fusion constructs to attempt to rescue the *Arabidopsis ttg2-1* mutant (Ler), which is characterized by fewer and unbranched trichomes, as well as a pale brown seed coat (Johnson *et al.*, 2002). These mutant phenotypes were rescued by introduction of the *35S:BnaA.TTG2.a.1:GFP* or *ProAtTTG2:AtTTG2:GFP* construct (Supplementary Fig. S4A–H, available at *JXB* online). We observed that other *Bna.TTG2* genes were also able to rescue the trichome defect in the *ttg2-1* mutant (Supplementary Fig. S4I–K). These results clearly demonstrated that *B. napus TTG2* genes function similarly to *AtTTG2* in leaf trichome development and in proanthocyanidin accumulation in the seed coat.

**Fig. 2.**
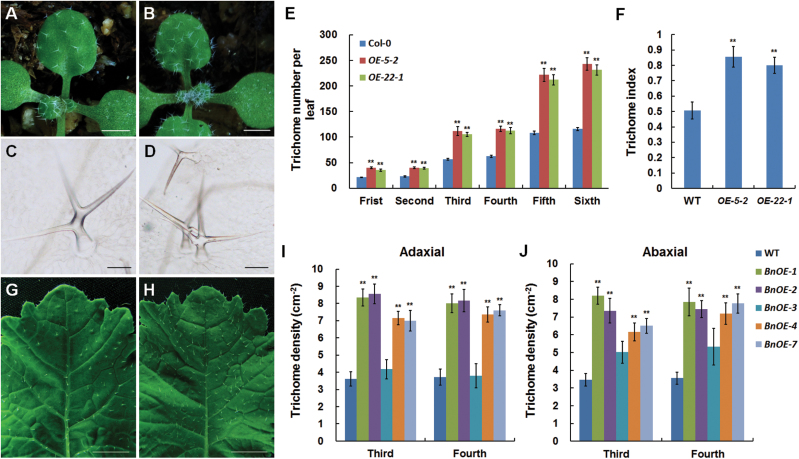
Trichome phenotypes of *35S:BnaA.TTG2.a.1:GFP* transgenic and WT *Arabidopsis* and *B. napus* plants. (A–D) Trichomes in 12-d-old WT *Arabidopsis* (A, C) and *35S:BnaA.TTG2.a:GFP* transgenic *Arabidopsis* (B, D). (E) Trichome numbers on the adaxial surfaces of the first six true leaves of WT and two *Arabidopsis* transgenic lines, *OE-5-2* and *OE-22-1*. (F) Trichome indices for the adaxial surfaces of the sixth true leaves of WT *Arabidopsis*, *OE-5-2*, and *OE-22-1* plants. (G, H) Trichomes on WT *B. napus* (G) and *35S:BnaA.TTG2.a:GFP* transgenic *B.napus* (H). (I, J) Trichome density on the adaxial (I) and abaxial (J) epidermis of the third and fourth true leaves of WT and *35S:BnaA.TTG2.a:GFP* transgenic *B. napus* plants. Bars, 1mm (A, B); 100 μm (C, D); 1cm (G, H). Data in (E), (F), (I), and (J) are presented as means±SE (*n*≥8) from three independent experiments. Asterisks (**) indicate a significant difference between WT and respective transgenic line at the *P*<0.01 level of the *t*-test.

Transgenic *Arabidopsis* plants overexpressing *BnaA.TTG2.a.1:GFP* (OE) showed increased trichome numbers (Fig. 2). To quantify the changes, two T3 transgenic lines, *OE-5-2* and *OE-22-1*, which contained single or two insertions and showed transgene expression (Supplementary Fig. S5A, B, available at *JXB* online), were used. Although no difference was observed in trichome morphology between the OE and WT plants (Fig. 2A, B), many trichomes in the OE plants developed in adjacent epidermal cells (Fig. 2C, D). The trichome numbers on the adaxial surfaces of the first six true leaves of the transgenic lines were almost twice those of WT (Fig. 2E), resulting in higher trichome indices in the transgenic lines (Fig. 2F). The phenotypes in OE plants were stable under a range of growing conditions with a temperature range of 18–22 °C and relative humidity from 45 to 60% under either a 16h day/8h night or 12h dat/12h night photoperiod. These results suggested that the overexpression of *BnaA.TTG2.a.1* affected both the initiation and spatial distribution of trichomes in *Arabidopsis* leaves.

To further validate the above observations, the *35S:BnaA.TTG2.a.1:GFP* construct was introduced into *B. napus*, and five transgenic lines were used to determine trichome phenotypes. The trichome numbers (expressed as density) on the adaxial and abaxial surfaces of the third and fourth true leaves of transgenic *Brassica* plants showed remarkable increases compared with WT (Fig. 2G–J), indicating that *BnaA.TTG2.a.1* also plays a similar role in trichome initiation in *B. napus*.

### *BnaA.TTG2.a.1*-overexpressing *Arabidopsis* and *B. napus* plants are hypersensitive to salt stress

**Fig. 3.**
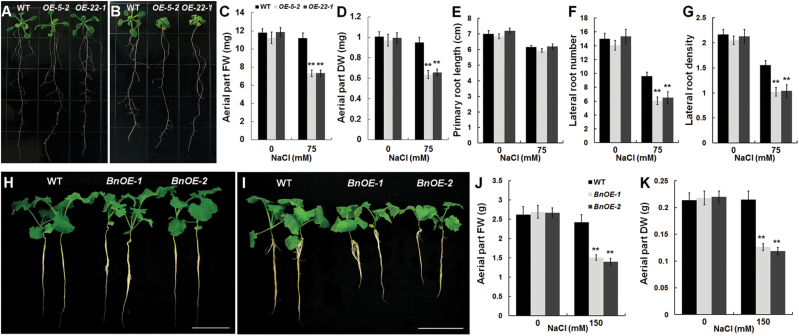
Response of *BnaA.TTG2.a.1*-overexpressing *Arabidopsis* and *B. napus* plants to salt stress. (A, B) Four-day-old WT and transgenic *Arabidopsis* plants (*OE-5-2* and *OE-22-1*) grown on ½ MS were transferred to ½ MS (A) or ½ MS containing 75mM NaCl (B) and allowed to grow for an additional 10 d. (C, D) Fresh weight (FW) (C) and dry weight (DW) (D) of the aerial part of WT and transgenic *Arabidopsis* plants treated with 75mM NaCl for 10 d (*n*=15). (E–G) Primary root length (E), lateral root number (F), and lateral root density (lateral roots per centimetre of the primary root) (G) of WT and transgenic *Arabidopsis* in ½ MS or ½ MS containing 75mM NaCl for 7 d (*n*=16–18). (H, I) WT and *35S:BnaA.TTG2.a.1:GFP B. napus* seedlings were grown with Hoagland solution (H) or Hoagland solution supplemented with 150mM NaCl (I) for 15 d. (J, K) FW (J) and DW (K) of the aerial part of WT and *35S:BnaA.TTG2.a.1:GFP* transgenic *B. napus* plants treated with Hoagland solution or Hoagland solution with 150mM NaCl for 15 d (*n*=10). Bars, 10cm (H, I). Data in (C–G), (J), and (K) are means±SE from three independent experiments. Asterisks (**) indicate a significant difference between WT and respective transgenic line at the *P*<0.01 level of the *t*-test.

Many WRKY TFs are involved in regulating plant abiotic stress (Rushton *et al.*, 2010), but such a role has not been reported for *TTG2* genes. To explore whether *BnaA.TTG2.a.1* is involved in plant responses to abiotic stress, the *Arabidopsis* homozygous T3 *BnaA.TTG2.a.1*-overexpressing lines *OE-5-2* and *OE-22-1* were subjected to salt stress. Without salt stress, no difference in growth between the OE and WT plants was observed (Fig. 3A). Surprisingly, the OE plants displayed dramatic growth inhibition (Fig. 3B) at 75mM NaCl with significantly reduced fresh and dry weights (Fig. 3C, D) compared with the WT plants. Both the number and density of the lateral roots in OE plants were reduced compared with those of WT in the presence of 75mM NaCl, although the OE roots exhibited a similar primary root length as the WT roots (Fig. 3E–G).

To confirm these *Arabidopsis* observations in *Brassica*, we analysed two *B. napus* transgenic lines, *BnOE-1* and *BnOE-2* (Supplementary Fig. S5C, D). In the absence of salt stress, the transgenic plants did not exhibit any notable differences in growth compared with the control plants (Fig. 3H). In contrast, the transgenic plants displayed hypersensitivity to salt stress (Fig. 3I). At 150mM NaCl, the fresh and dry weights of the aerial parts of the transgenic plants were significantly lower than those of the WT plants (Fig. 3J, K). These results demonstrated that *BnaA.TTG2.a.1* overexpression enhanced salt sensitivity in transgenic *B. napus*, consistent with the observation in *Arabidopsis*.

**Fig. 4.**
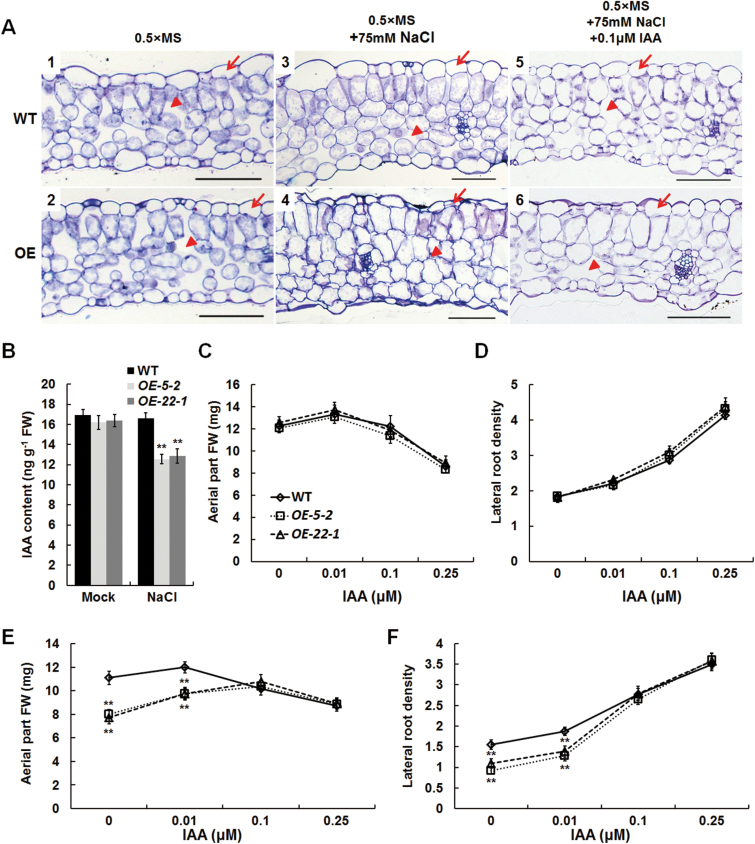
Leaf cellular structure and endogenous IAA level in *BnaA.TTG2.a.1* transgenic and WT *Arabidopsis* plants. (A) Cross-sections of leaves of WT *Arabidopsis* (panels 1, 3, and 5) and representative *BnaA.TTG2.a.1* OE (panels 2, 4, and 6) plants in ½ MS, ½ MS containing 75mM NaCl, and ½ MS containing 75mM NaCl and 0.1 µM IAA, respectively. WT and OE plants were treated for 7 d, and the sixth true leaves were used for the analysis. The OE plants showed thinner epidermal cells (arrows), shrunken mesophyll cells and decreased intercellular space (arrow heads) compared with WT plants under salt stress. (B) IAA contents in WT and *BnaA.TTG2.a.1* OE *Arabidopsis* seedlings under normal and salt stress conditions. Seeds were germinated on ½ MS medium for 4 d and seedlings were transferred to ½ MS or ½ MS containing 75mM NaCl for 3 d (*n*≥4). IAA levels were determined by liquid chromatography/mass spectrometry analysis. (C, D) Fresh weight of the aerial part (C) and lateral root density (D) of transgenic plants grown in the presence of IAA. (E, F) Fresh weight of the aerial part (E) and lateral root density (F) of transgenic plants grown in the presence of NaCl and IAA. Four-day-old seedlings grown on ½ MS were transferred to ½ MS supplemented with IAA (0, 0.01, 0.1, or 0.25 μM) or supplemented with both 75mM NaCl and IAA (0, 0.01, 0.1, or 0.25 μM) for another 10 d; the lateral root density was measured on day 7, and the aerial part (FW) was measured on day 10 (*n*≥14). Bars, 20 μm (A). Data in (B)–(F) are means±SE from three biologically independent experiments. Asterisks indicate statistically significant differences (*P<0.05, **P<0.01; *t*-test). (This figure is available in colour at *JXB* online.)

As the leaves of the OE *Arabidopsis* plants grown under salt stress showed more downward curling than the WT leaves (Fig. 3B), the cellular structure was analysed by semi-thin sectioning and light microscopy. Under normal conditions, similar cell layers and cell morphology were observed in the leaves of both WT and OE plants (Fig. 4A, panels 1 and 2). Under salt stress, the mesophyll cells in the WT and OE leaves were larger than their respective counterparts under normal conditions, consistent with a previous study on *Aster tripolium* under saline conditions (Geissler *et al.*, 2009). However, the OE leaves had visibly thinner epidermal cells, shrunken mesophyll cells, and decreased intercellular space in comparison with the WT leaves under saline conditions (Fig. 4A, panels 3 and 4). Therefore, the moderate level of salt stress affected the cellular morphology in the leaves of OE but not WT plants.

### The endogenous IAA level is reduced in *BnaA.TTG2.a.1*-overexpressing plants under salt stress

To investigate the possible causes of the salt sensitivity of OE plants, we examined several parameters frequently used in salt stress studies. First, the aerial part fresh and dry weights and lateral root numbers of the OE plants were found to be similar to those of the WT plants grown on ½ MS medium with 300mM mannitol as an osmolyte (Supplementary Fig. S6A–D, available at *JXB* online), indicating that osmotic stress is not the major factor responsible for the salt sensitivity observed in OE plants. Secondly, Na^+^ and K^+^ content in both transgenic *Arabidopsis* and *B. napus* seedlings, as well as in the respective WT, were determined under normal and salt-stressed conditions. The Na^+^ and K^+^ contents and Na^+^/K^+^ ratios of both transgenic *Arabidopsis* and *B. napus* were similar to those of WT (Supplementary Fig. S6E–J), suggesting that the salt hypersensitivity of the OE plants was not caused by disruption of Na^+^ and K^+^ homeostasis. Thirdly, OE and WT plants were treated with various concentrations of exogenous ABA. At all of the tested concentrations, the growth performances and transcript levels of several ABA-responsive genes showed no significant difference between the OE and WT plants (Supplementary Fig. S7, available at *JXB* online), indicating that the salt sensitivity of the transgenic plants was not mediated by ABA.

The phenotypes displayed by OE plants, such as dwarfed shoots, curled leaves, and reduced lateral root numbers (Fig. 3), suggested the possible involvement of auxin. We thus determined the free IAA levels in the plants. There was no obvious difference in the free IAA content between the OE and WT plants under normal growth conditions (Fig. 4B, Mock; Supplementary Table S2, available at *JXB* online), whereas the free IAA content in the OE plants grown at 75mM NaCl was significantly lower than that in the WT plants (Fig. 4B, NaCl; Supplementary Table S2).

To analyse the relationship of IAA and salt stress further, exogenous IAA was applied to plants with or without NaCl. Without salt stress, there was no significant difference in the shoot fresh weights and lateral root density between the WT and OE plants at any exogenously applied IAA concentration (Fig. 4C, D). Under 75mM NaCl, however, the OE plants had a lower shoot weight and fewer lateral roots than the WT plants (Fig. 3A–G; Fig. 4E, F). The differences between the OE and WT plants were gradually reduced when an increasing concentration of IAA was used; at 0.10 and 0.25 μM IAA, these differences were greatly diminished (Fig. 4E, F), and the OE seedlings also exhibited normal cellular morphology under salt stress (Fig. 4A, panels 5 and 6). These observations clearly showed that the addition of exogenous IAA to the NaCl-containing medium could alleviate the hypersensitivity of the OE plants to salt stress. Together with the observation that the OE plants under salt stress had a reduced level of IAA (Fig. 4B), our results suggest that the salt stress sensibility displayed by the *BnaA.TTG2.a.1*-overexpressing plants was probably due to a lower level of IAA.

### Expression of auxin biosynthesis- and auxin response-related genes is altered in *BnaA.TTG2.a.1*-overexpressing *Arabidopsis*

**Fig. 5.**
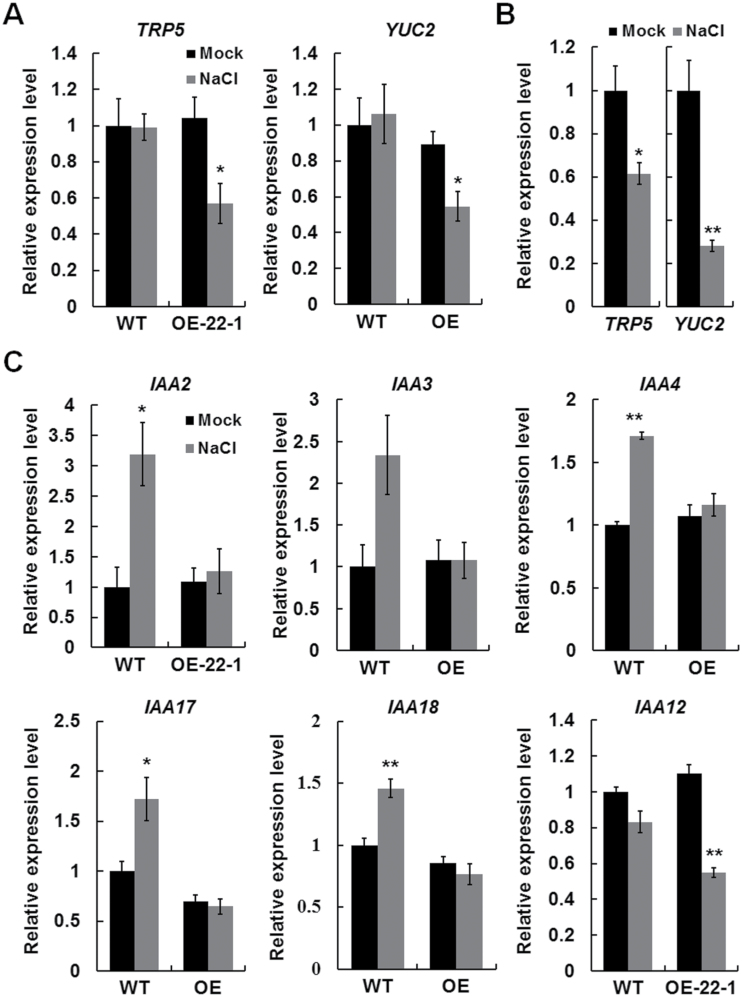
Expression of auxin synthesis and response genes in *BnaA.TTG2.a.1*-overexpressing and WT *Arabidopsis* plants. (A) Relative expression of the auxin biosynthesis genes *TRP5* and *YUC2* in WT and OE *Arabidopsis* plants treated with or without 75mM NaCl. (B) Relative expression of *TRP5* and *YUC2* in WT *Arabidopsis* treated with 125mM NaCl. (C) Relative expression of auxin response genes in WT and OE plants treated with or without 75mM NaCl. Data in (A)–(C) are means±SE from three biologically independent experiments. Asterisks indicate statistically significant differences (*P<0.05, **P<0.01; *t*-test).

The reduced IAA levels in the OE plants under salt stress could be due to either reduced biosynthesis or increased metabolism. *TRP5* and *YUC2* are two important auxin biosynthesis genes in *Arabidopsis* (Stepanova *et al.*, 2005; Mashiguchi *et al.*, 2011), and the expression profiles of *TRP5* and *YUC2* overlap with that of *AtTTG2* in several tissues (Supplementary Fig. S8, available at *JXB* online). Furthermore, *TRP5* and *YUC2* were found to be downregulated in the OE plants at 75mM NaCl but not in the WT plants (Fig. 5A). To determine whether a higher threshold of salt stress is needed for downregulating *TRP5* and *YUC2* in WT, we investigated their expression in WT plants at 125mM NaCl and found that the expression of both genes was significantly downregulated (Fig. 5B). These results showed that salt stress downregulated the expression of *TRP5* and *YUC2* and that this effect was enhanced by *BnaA.TTG2.a.1* overexpression.

We further analysed the expression of several auxin response genes in OE and WT plants under salt stress. The transcript levels of *IAA2*, *IAA3*, *IAA4*, *IAA17*, and *IAA18* were upregulated in WT plants following 75mM NaCl treatment for 48h, whereas no significant changes in expression were observed in OE plants. In addition, the expression of *IAA12* was not affected by salt stress in the WT plants, whereas it was significantly downregulated in the OE plants under salt stress (Fig. 5C). These results indicated that overexpressing *BnaA.TTG2.a.1* in *Arabidopsis* suppressed the expression of certain auxin response genes.

### BnaA.TTG2.a.1 binds to the W-box of the *TRP5* and *YUC2* promoters

**Fig. 6.**
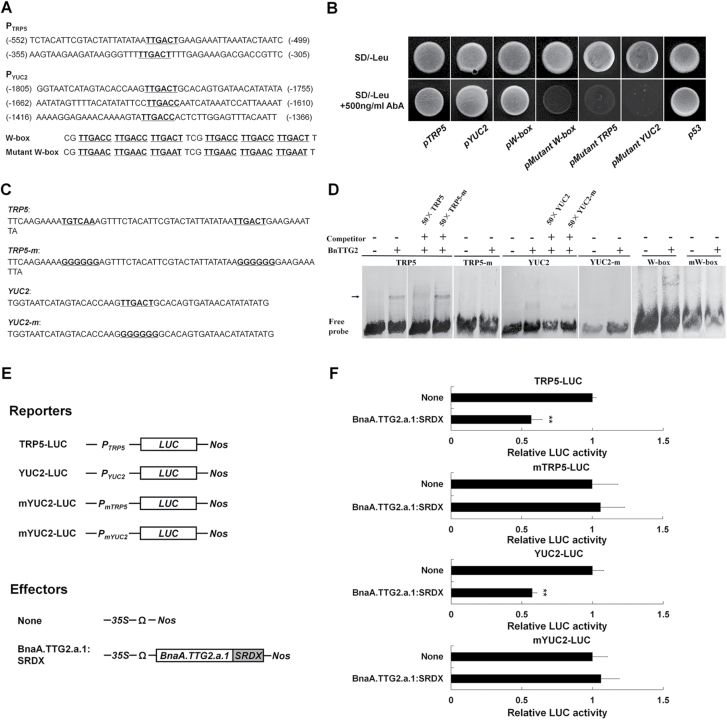
Yeast one-hybrid, electrophoretic mobility shift, and DLR assays for binding of BnaA.TTG2.a.1 to the promoters of *TRP5* and *YUC2*. (A) Nucleotide sequences of regions of the *TRP5* and *YUC2* promoters containing putative W-box motifs (bold and underlined). The W-box and mutant W-box shown below the promoter sequences were used as the positive and negative control, respectively. (B) Growth of the Y1HGold yeast strain in the absence (top) and presence (bottom) of 500ng ml^–1^ of AbA on SD–Leu plates. (C) Nucleotide sequences containing the W-boxes or mutated W-boxes in the promoter region of *TRP5* and *YUC2* (bold and underlined). (D) Binding of BnaA.TTG2.a.1 to the *TRP5* and *YUC2* promoter fragments. Unlabelled promoter fragments were used as competitor for the assays. Arrows highlight the shifted bands. (E) Schematic representation of the reporter constructs used for transient assays in *Arabidopsis* mesophyll protoplasts. For each reporter construct, the firefly *LUC* gene is driven by the *TRP5*, *YUC2*, mutated *TRP5* (*TRP5-m*), or mutated *YUC2* (*YUC2-m*) promoter. BnaA.TTG2.a.1 fused with a chimaeric repression domain (SRDX) and driven by the 35S promoter plus the translation enhancer Ω was used as an effector. The empty vector (None) was used as a control, and the *Renilla LUC* gene driven by the *AtUBI3* promoter was used as an internal control. (F) Relative *LUC* activities detected in the transient expression assays (*n*=5). Error bars represent the SE of three biological replicates, and the value of the control was set to 1. Asterisks (**) indicate a significant difference between the WT and respective transgenic line at the *P*<0.01 level as determined by a *t*-test.

The reduced expression levels of *TRP5* and *YUC2* in OE plants under salt stress indicated the possibility that *BnaTTG2* genes may be involved in the direct regulation of their expression in response to salt stress. It is known that WRKY TFs bind specifically to W-boxes in their target genes (Rushton *et al.*, 2010), and sequence analysis identified putative W-boxes in both the *TRP5* and *YUC2* promoter regions (Fig. 6A). To determine whether Bna.TTG2 proteins bind directly to the *TRP5* and *YUC2* promoters, particularly the W-box, we performed an Y1H assay with WT and mutated *TRP5* and *YUC2* promoters (Fig. 6A). Yeast cells harbouring each of these Y1H constructs grew on SD–Leu medium. Yeast cells co-transformed with *p53-AbAi* and *BnaA.TTG2.a.1-pGADT7* (the positive control) grew normally on SD–Leu medium and SD–Leu medium with AbA (SD–Leu+AbA) (Fig. 6B, far-right column). Similarly, yeast cells co-transformed with *BnaA.TTG2.a.1-GADT7* and *pTRP5-AbAi*, *pYUC2-AbAi*, or *pW-box-AbAi* grew as well as the positive control on SD–Leu+AbA medium (Fig. 6B, first three columns), suggesting that BnaA.TTG2.a.1 can bind to the W-box in the promoters of both *TRP5* and *YUC2* genes in yeast cells. In contrast, the yeast cells co-transformed with *BnaA.TTG2.a.1-GADT7* and *pmW-Box-pAbAi*, *pmTRP5-AbAi*, or *pmYUC2-AbAi* (with the W-box sequences in the promoters changed from TTGACT/C to TTGAAT/C) failed to grow on SD–Leu+AbA medium (Fig. 6B, fourth to sixth columns). These results thus strongly indicated that BnaA.TTG2.a.1 can bind to the W-box in the promoters of both *TRP5* and *YUC2* genes in yeast cells.

To further confirm the above observations, an electrophoretic mobility shift assay was performed. Our results showed that BnaA.TTG2.a.1 was able to bind to a DNA fragment containing a W-box located in the –514 to –572bp region of the *TRP5* promoter, one in the –1759 to –1806-bp region of the *YUC2* promoter, and the classical W-box. Conversely, BnaA.TTG2.a.1 failed to bind to the W-box-mutated fragments of the *TRP5* and *YUC2* promoters and the mutated W-box (Fig. 6C, D). These results thus provided further support that both *TRP5* and *YUC2* are direct targets of BnaA.TTG2.a.1.

A previous study showed that fusion of AtTTG2 with the modified EAR motif repression domain SRDX (Hiratsu *et al.*, 2003) (*ProAtTTG2:AtTTG2:SRDX*) could suppress *AtTTG2* function with regard to the transcription of its target genes in *Arabidopsis* (Ishida *et al.*, 2007). We therefore tested the interaction of BnaA.TTG2.a.1 and the *TRP5* and *YUC2* promoters in plant cells by a DLR assay using *Arabidopsis* mesophyll protoplasts. The coding sequence of *BnaA.TTG2.a.1* was fused with *SRDX* and placed behind the 35S promoter, and the empty vector was used as a control (Fig. 6E). When *BnaA.TTG2.a.1:SRDX* was not present, all the reporter constructs of *TRP5–LUC*, *mTRP5–LUC*, *YUC2–LUC*, and *mYUC2–LUC* exhibited similar activity (Fig. 6E, F). Compared with the vector control, *BnaA.TTG2.a.1:SRDX* strongly impaired the LUC activity of the *TRP5* and *YUC2* reporters (~50% reduction), whereas such a reduction was not observed with the *mTRP5* and *mYUC2* reporters (Fig. 6F). These results demonstrated that BnaA.TTG2.a.1 was able to directly bind to the W-box of the *TRP5* and *YUC2* promoters in plant cells.

### The N-terminal region of BnaA.TTG2.a.1 may play an important role in salt stress responses

**Fig. 7.**
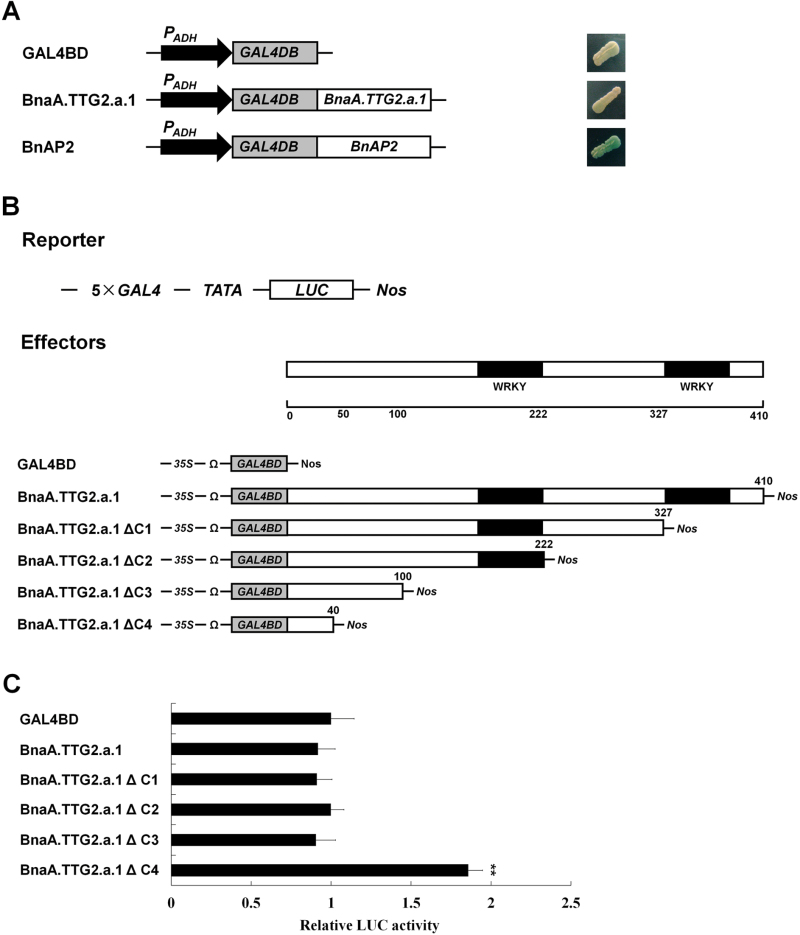
Transcriptional activation analysis of BnaA.TTG2.a.1. (A) Transcriptional activation analysis of full-length BnaA.TTG2.a.1 in yeast. On the left is shown a schematic of the constructs used for the assay. BnaA.TTG2.a.1 and BnAP2 were fused with GAL4BD. The GAL4BD empty vector was used as a negative control, and the BnAP2 protein was used as a positive control. The transactivation activity assay was conducted with the yeast strain MaV203. The right column shows the results of the yeast transactivation assay on SC–Leu–His medium containing 40 μg ml^–1^ of X-α-gal. (B) Schematic representation of the constructs used in the *Arabidopsis* mesophyll protoplast transient assay. The reporter construct GAL4–LUC contains five copies of the GAL4 responsive element, the minimal TATA box region of the 35S promoter, the firefly *LUC* gene and a NOS terminator. The full-length BnaA.TTG2.a.1 and different truncated segments of BnaA.TTG2.a.1 were fused with GAL4BD driven by the 35S promoter plus the transcriptional enhancer element Ω. GAL4BD was used as a negative control. (C) The transcriptional activation abilities of full-length BnaA.TTG2.a.1 and different fragments of BnaA.TTG2.a.1 are indicated by the relative LUC activities of the reporter. Error bars represent the SE of three biological replicates, and the value of the negative control was set to 1 (*n*=5). Asterisks (**) indicate a significant difference between WT and respective transgenic line at the *P*<0.01 level of the *t*-test. (This figure is available in colour at *JXB* online.)

AtTTG2 is classified as a member of the WRKY TF family (Johnson *et al.*, 2002); however, its transactivation activity has not been determined directly. To better understand the structure–function relationship of Bna.TTG2 proteins, the ability of full-length BnaA.TTG2.a.1 to activate gene expression was analysed in yeast. A previously reported TF, BnAP2 (Yan *et al.*, 2012), was used as a positive control, and the empty vector GAL4BD was used as a negative control. Yeast cells containing BnAP2–GAL4BD exhibited a blue colour on SC–Leu–His medium with 40 μg X-α-gal ml^–1^. However, the cells containing full-length BnaA.TTG2.a.1–GAL4BD or the negative control GAL4BD did not produce a blue colour (Fig. 7A), indicating the absence of transcriptional activation by full-length BnaA.TTG2.a.1 in this assay.

A possible explanation for the above results is that a repressor domain might be present in the BnaA.TTG2.a.1 protein. To examine this possibility, a DLR assay system using *Arabidopsis* mesophyll protoplasts (Hao *et al.*, 2011) was performed with full-length BnaA.TTG2.a.1 and a series of BnaA.TTG2.a.1 deletions (Fig. 7B). Compared with the negative control GAL4BD, full-length BnaA.TTG2.a.1 and three C-terminal deletions (BnaA.TTG2.a.1ΔC1, BnaA.TTG2.a.1ΔC2, and BnaA.TTG2.a.1ΔC3) were unable to activate the reporter gene. Interestingly, BnaA.TTG2.a.1ΔC4 (containing the first 40 aa of BnaA.TTG2.a.1) could activate the reporter gene, resulting in an almost doubled LUC activity compared with the negative control GAL4BD (Fig. 7C). The results suggested the possible presence of a repressor domain in the region of residues 41–100.

**Fig. 8.**
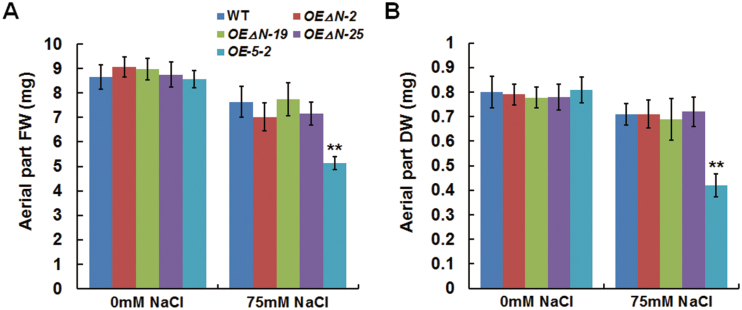
Salt tolerance of *BnaA.TTG2.a.1△N*-overexpressing *Arabidopsis*. (A) Fresh (FW) and (B) dry weights (DW) of the aerial part of WT, *OE-5-2* and three independent lines of *BnaA.TTG2.a.1△N*-overexpressing (lacking the N-terminal 77 aa) *Arabidopsis* (*OE△N*) plants treated with 75mM NaCl. Four-day-old WT and transgenic *Arabidopsis* plants grown on ½ MS were transferred to ½ MS with or without 75mM NaCl and allowed to grow for an additional 10 d (n≥10). Data in (A and B) are the means±SE from three biologically independent experiments. Asterisks indicate statistically significant differences (**P<0.01, *t*-test).

Overexpression of full-length *BnaA.TTG2.a.1* suppressed the expression of auxin biosynthesis-related genes under salt stress (Fig. 5A), suggesting that BnaA.TTG2.a.1 might act as a transcriptional repressor under salt stress. To understand the importance of the N-terminal region of BnaA.TTG2.a.1 in salt stress responses, we generated a BnaA.TTG2.a.1 deletion construct lacking the N-terminal 77 aa and fused with GFP (named *BnaA.TTG2.a.1△N:GFP*). Three T3 homozygous *Arabidopsis* lines derived from the above deletions of *BnaA.TTG2.a.1* (*OEΔN-2*, *-19*, and *-25*) together with WT and *OE-5-2* plants were grown under salt stress. The fresh and dry weights of *OE-5-2* were significantly lower than those of WT under salt stress, whereas no significant differences were observed between the *OEΔN* and WT plants under the same conditions (Fig. 8). These results suggested that removal of the N-terminal region had diminished the ability of BnaA.TTG2.a.1 to confer salt stress sensitivity to transgenic plants.

## Discussion

### Overexpression of *BnaA.TTG2.a.1* results in reduced IAA levels and inhibits the growth of transgenic plants under salt stress

The majority of WRKY TFs characterized to date are involved in various abiotic stress responses (Chen *et al.*, 2012; Rushton *et al.*, 2012). As a member of the *Arabidopsis* WRKY family, AtTTG2 was originally identified as being involved in trichome initiation, proanthocyanidin accumulation in the seed coat and seed development (Johnson *et al.*, 2002; Ishida *et al.*, 2007; Dilkes *et al.*, 2008), but there is no evidence for its involvement in any stress response. In this study, we showed that transgenic *Arabidopsis* and *Brassica* plants overexpressing *BnaA.TTG2.a.1* were hypersensitive to salt stress, thus revealing a novel function of *TTG2* genes.

Excessive Na^+^ accumulation in cells is regarded as the primary cause of plant growth inhibition under salt stress (Ghoulam *et al.*, 2002; Türkan and Demiral, 2009). Ion homeostasis in plant cells and plant responses specific to ion toxicity are regulated by the slat overly sensitive (SOS) pathway (Hasegawa *et al.*, 2000; Zhu, 2002). However, we did not observe any difference in ion content or homeostasis between OE and WT plants (Supplementary Fig. S6E–J), suggesting that BnaA.TTG2.a.1 may negatively regulate plant salt stress responses through an SOS-independent pathway. Furthermore, osmotic stress contributes to slower plant growth under salt stress (Martınez-Ballesta *et al.*, 2004). We analysed the possible contribution of osmotic pressure and determined that it was not the main reason for the salt sensitivity phenotype of OE plants (Supplementary Fig. S6A–D). Moreover, ABA is a plant hormone that coordinates responses to stresses, including salt stress (Umezawa *et al.*, 2010), and our results suggested that the salt sensitivity of OE plants was not mediated by ABA (Supplementary Fig. S7). Therefore, the salt sensitivity displayed by *BnaA.TTG2.a.1*-overexpressing plants might result from mechanisms other than ionic, osmotic stress, or ABA-mediated pathways.

The phenotypes of *BnaA.TTG2.a.1*-overexpressing *Arabidopsis* plants under salt treatment, such as dwarfed shoots, curled leaves, and reduced plant growth, mimicked the phenotypic changes of auxin deficiency in *Arabidopsis* and other plants. We further showed that *BnaA.TTG2.a.1*-overexpressing *Arabidopsis* plants had a significantly lower level of IAA compared with WT plants (Fig. 4B), and that the growth and cellular morphology phenotypes of OE plants under salt stress could be reversed by the application of exogenous auxin (Fig. 4E, F), consistent with previous studies in *Arabidopsis* and other species (Takase *et al.*, 2004; Park *et al.*, 2007). These data strongly indicated that a lower IAA level is a main factor contributing to the salt-sensitive phenotype of *BnaA.TTG2.a.1*-overexpressing plants.

### BnaA.TTG2.a.1 may affect IAA levels largely through the regulation of *TRP5* and *YUC2* genes

*TRP5* and *YUC2* are involved in the tryptophan-dependent IAA biosynthesis pathway (Mano and Nemoto, 2012). *TRP5* regulates an important step in the tryptophan biosynthesis pathway, and loss of *TRP5* function results in defective tryptophan biosynthesis, leading to reduced endogenous IAA levels (Stepanova *et al.*, 2005). *YUC2* encodes a flavin mono-oxygenase-like protein that catalyses the conversion of indole-3-pyruvic acid to IAA (Mashiguchi *et al.*, 2011). In OE plants, the expression of *TRP5* and *YUC2* was significantly downregulated, and the IAA concentration was reduced under salt stress (Figs 5A and 4B). Comprehensive analyses showed that *BnaA.TTG2.a.1* can bind to the promoters of *TRP5* and *YUC2* (Fig. 6). However, a higher concentration of NaCl (125mM) was needed to suppress the expression of *TRP5* and *YUC2* in WT (Fig. 5B), suggesting that *BnaA.TTG2.a.1* may act as a transcriptional suppressor of *TRP5* and *YUC2* under salt stress in a dose-dependent manner.

The *AUX*/*IAA* gene family includes a group of early auxin response genes encoding short-lived nuclear proteins that act as transcriptional repressors of auxin-responsive reporter genes (Abel and Theologis, 1996). These repressors prevent ARF transcriptional activators from reaching their target sites and play important roles in regulating auxin-induced gene expression and plant development (Leyser, 2002; Kieffer *et al.*, 2010). In this study, we found that several *AUX*/*IAA* genes, *IAA2*, *IAA3*, *IAA4*, *IAA17*, and *IAA18*, were upregulated in WT *Arabidopsis* under 75mM NaCl (Fig. 5C), suggesting that salt stress affects the auxin signalling pathway. This observation was consistent with a previous study that reported the upregulated expression of certain *AUX*/*IAA* genes upon salt stress in WT *Arabidopsis* (Jiang and Deyholos, 2006). Nonetheless, at this level of salt stress, the expression of *TRP5* and *YUC2* and the IAA content in WT plants were not significantly different from the WT plants grown under normal conditions (Fig. 5A). Taken together, these data indicated that the upregulation of *AUX*/*IAA* genes in WT to the extent observed in this study was not sufficient to affect the endogenous IAA level under such a salt stress. In contrast, a significant decline in IAA content in *BnaA.TTG2.a.1*-overexpressing plants was found under 75mM NaCl, in accordance with their salt-hypersensitive phenotypes (Figs 3 and 4). The above observation indicates that plants have the ability to maintain a balance to adapt to stress by regulating gene expression under salt stress. Such an adaptation may also involve the adjustment of auxin levels, as a previous study found that mild salt stress promotes auxin accumulation in developing *Arabidopsis* lateral root primordia, preventing their developmental arrest under this stress (Zolla *et al.*, 2010). An enhanced level of *TTG2* gene expression in OE plants can result in the downregulation of *TRP5* and *YUC2* and thus break such a balance (Figs 4B and 5A). At the same time, the expression of other IAA pathway genes was not upregulated; as a result, the IAA content declined. WT plants did not show significantly downregulated expression of *TRP5* and *YUC2* and could maintain a more normal growth than the OE plants under 75mM NaCl (Fig. 5A). Together, these observations indicated that the maintenance of a normal level of *Bna.TTG2* gene expression is required for the plant to adapt to salt stress by retaining the normal expression levels of *TRP5* and *YUC2*, and thus a stable IAA level.

### Bna.TTG2 proteins act as dual-functional TFs in trichome development and salt stress responses

A number of TFs possess both transcriptional activation and repression domains and act as either transcriptional repressors or activators. Interestingly, some TFs act as a transcriptional activator in one pathway and as a transcriptional repressor in another (Ikeda *et al.*, 2009). In this regard, increasing evidence suggests that certain WRKY proteins function in multiple biological pathways. For example, *Arabidopsis AtWRKY33* positively regulates jasmonic acid- and ethylene-mediated plant resistance responses, and *AtWRK33* mutant plants exhibit enhanced susceptibility to necrotrophic fungal pathogens (Zheng *et al.*, 2006). However, the expression of *AtWRKY33* also partially depends on ABA biosynthesis and signalling, and *AtWRKY33* overexpression in *Arabidopsis* increases tolerance to salt stress (Jiang and Deyholos, 2009).

The mechanisms by which a TF can function in two or more diverse biological pathways are still not clear. TFs usually possess the functional property of transcriptional activation or repression by binding to specific DNA sequences. In our study, *Bna.TTG2* genes were found to have the ability to rescue the trichome number and branching phenotypes of the *Arabidopsis ttg2-1* mutant (Supplementary Fig. S4), demonstrating that the Bna.TTG2 proteins function as a transcriptional activator in trichome development in a manner similar to that of AtTTG2 (Ishida *et al.*, 2007). In addition, BnaA.TTG2.a.1 may also function as a transcriptional repressor under salt stress conditions by binding directly to the promoters of auxin biosynthesis genes. These results suggest that Bna.TTG2 proteins participate in both developmental biology and stress response pathways and may act as dual-function TFs.

Auxin plays important roles in trichome initiation and development (Zhang *et al.*, 2011; Deng *et al.*, 2012) and in plant responses to salt stress (Dunlap and Binzel, 1996; Wang *et al.*, 2009; Zorb *et al.*, 2013). Previous studies have shown that trichome number is not responsive to exogenous auxin application (Kim *et al.*, 2007), yet is affected by the location and timing of auxin accumulation (Zhang *et al.*, 2011). Regardless, the growth of root hairs is promoted by auxin (Rahman *et al.*, 2002; Ishida *et al.*, 2008). A recent study also showed that GhTCP14, a cotton TF involved in auxin-mediated trichome formation, is able to positively or negatively regulate the expression of auxin response and transporter genes as a dual-function TF(Wang *et al.*, 2013). In our study, the overexpression of *BnaA.TTG2.a.1* increased the trichome number (Fig. 2). However, it was not clear whether this increased trichome initiation in OE plants was related to changes in the location or timing of IAA accumulation in leaves. In our study, the IAA levels were similar between WT and OE plants under normal conditions (Fig. 4B), at which the OE plants developed more trichomes (Fig. 2). A decreased IAA level was detected in OE plants only under salt stress and was accompanied by a decrease in lateral root numbers (Fig. 3F, G). *BnaA.TTG2.a.1* could bind to the *TRP5* and *YUC2* promoters (Fig. 6), reducing the transcription of these genes and the biosynthesis of IAA. The mechanism by which *Bna.TTG2* genes function in trichome development and salt stress responses warrants further study. We showed that removal of the N-terminal region could reduce the ability of BnaA.TTG2.a.1 to confer salt stress sensitivity to transgenic plants (Fig. 8). Such an observation indeed provides a clue for the screening of co-factors that can interact with the N terminus of BnaA.TTG2.a.1. Further elucidation of the connections among *Bna.TTG2* genes, auxin, trichome development, and salt stress responses based on the current findings will help to gain a better understanding of how WRKY TFs function in different biological processes, which in turn could allow the fine manipulation of genes with broad-spectrum effects on various stress responses.

## Supplementary data

Supplementary data are available at *JXB* online.

Supplementary Fig. S1. Southern blotting analysis of *Bna.TTG2* genes in *B. rapa*, *B. oleracea* and *B. napus*.

Supplementary Fig. S2. Sequence analysis of the *Bna.TTG2* gene family from *B. napus*.

Supplementary Fig. S3. Subcellular localization of *B. napus* TTG2 proteins.

Supplementary Fig. S4. Complementation of the *Arabidopsis ttg2* mutant by *B. napus TTG2* genes.

Supplementary Fig. S5. Copy number and expression analysis of *35S:BnaA.TTG2.a.1:GFP* in transgenic *Arabidopsis* and *B. napus*.

Supplementary Fig. S6. Effects of osmotic stress and ionic toxicity on plant growth in *BnaA.TTG2.a.1*-overexpressing *Arabidopsis* and *B. napus*.

Supplementary Fig. S7. Effects of ABA on plant growth in *BnaA.TTG2.a.1*-overexpressing *Arabidopsis*.

Supplementary Fig. S8. Relative expression levels of *TRP5* and *YUC2* genes and *AtTTG2* in different tissues in *Arabidopsis*.

Supplementary Methods. Genomic DNA isolation and Southern analysis, ion content determination and plasmid preparation.

Supplementary Table S1. List of primers used in this study.

Supplementary Table S2. Measurements of endogenous IAA contents in WT and OE plants under the conditions with or without salt stress.

Supplementary Data

## Abbreviations:

ABA: abscisic acid
AbA: aureobasidin A
DLR: dual-luciferase reporter
GFP: green fluorescent protein
GUS: β-glucuronidase
HS: Hoagland nutrient solution
IAA: indole-3-acetic acid
LUC: luciferase
½ MS: half-strength Murashige and Skoog medium
OE: overexpression
qRT-PCR: quantitative real-time PCR
SOS: salt overly sensitive
TF: transcription factor
WT: wild type
Y1H: yeast one-hybrid.
