# Supplementary material for: Expression of Brassica napus TTG2, a regulator of trichome development, increases plant sensitivity to salt stress by suppressing the expression of auxin biosynthesis genes
Source: J Exp Bot. 2015 Jun 12;66(19):5821–36. doi: 10.1093/jxb/erv287 (PMC4566978; doi:10.1093/jxb/erv287)
Supplement: Supplementary Data [file supp_66_19_5821__index.html]

Expression of Brassica napus TTG2, a regulator of trichome development, increases plant sensitivity to salt stress by suppressing the expression of auxin biosynthesis genes — Expression of Brassica napus TTG2, a regulator of trichome development, increases plant sensitivity to salt stress by suppressing the expression of auxin biosynthesis genes — Supplementary Data 

# Expression of *Brassica napus TTG2*, a regulator of trichome development, increases plant sensitivity to salt stress by suppressing the expression of auxin biosynthesis genes

## Supplementary Data

Data files

- Supplementary Data - Supplementary Data
