# Supplementary material for: Expression of Brassica napus TTG2, a regulator of trichome development, increases plant sensitivity to salt stress by suppressing the expression of auxin biosynthesis genes
Source: J Exp Bot. 2015 Jun 12;66(19):5821–36. doi: 10.1093/jxb/erv287 (PMC4566978; doi:10.1093/jxb/erv287)
Supplement: Supplementary Data [file supp_erv287_jexbot145870_file001.pdf]

Supplementary Data

Manuscript Title

Trichome development regulator TTG2 of Brassica napus confers the plant sensitivity to salt stress by suppressing expression of auxin biosynthesis genes

Qingyuan Li, Mei Yin, Yongpeng Li, Chuchuan Fan, Qingyong Yang, Jian Wu, Chunyu Zhang, Hong Wang, Yongming Zhou*

Supplementary Figures:

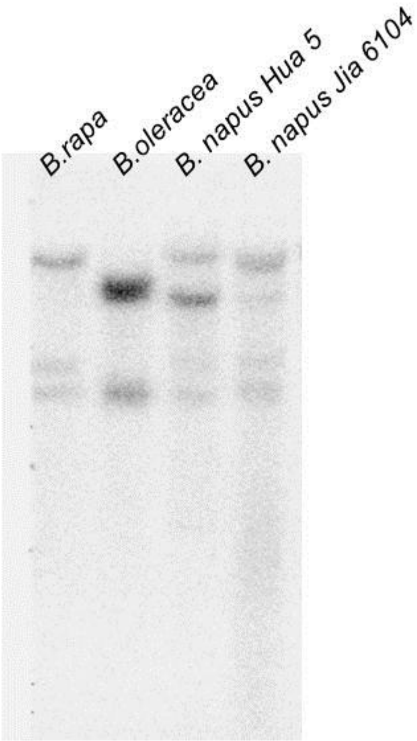

Figure S1. Southern blotting analysis of *Bna.TTG2* genes in *B. rapa*, *B. oleracea* and *B. napus*.

The genomic DNA was digested with *Pat*I before electrophoresis.

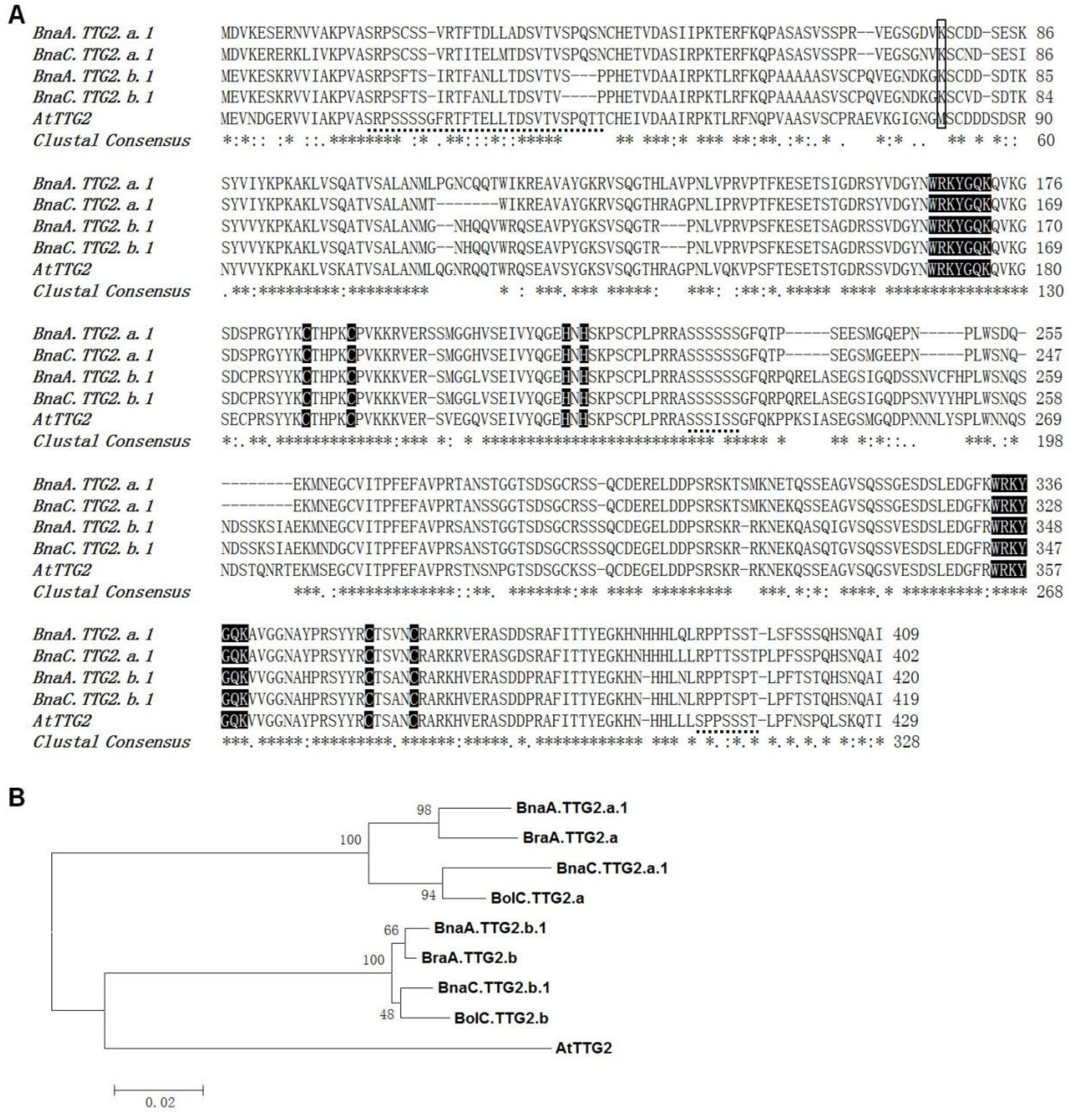

Figure S2. Sequence analysis of the *Bna.TTG2* gene family from *B. napus*.

(A) Sequence alignment of the deduced Bna.TTG2 sequences with AtTTG2 (AT2G37260.1). WRKY domains are indicated by white letters on a black background. The black box indicates the corresponding site of translation start codon of the splicing variant AT2G37260.2 in Arabidopsis. The putative Ser/Thr-rich activation domains according to a previous study (Johnson *et al.*, 2002) are highlighted (dotted underline). (B) Phylogenetic relationships of the TTG2 proteins from B. rapa, B. oleracea, B. napus and Arabidopsis.

Phylogenetic analysis was performed with the neighbor-joining method using MEGA 5.

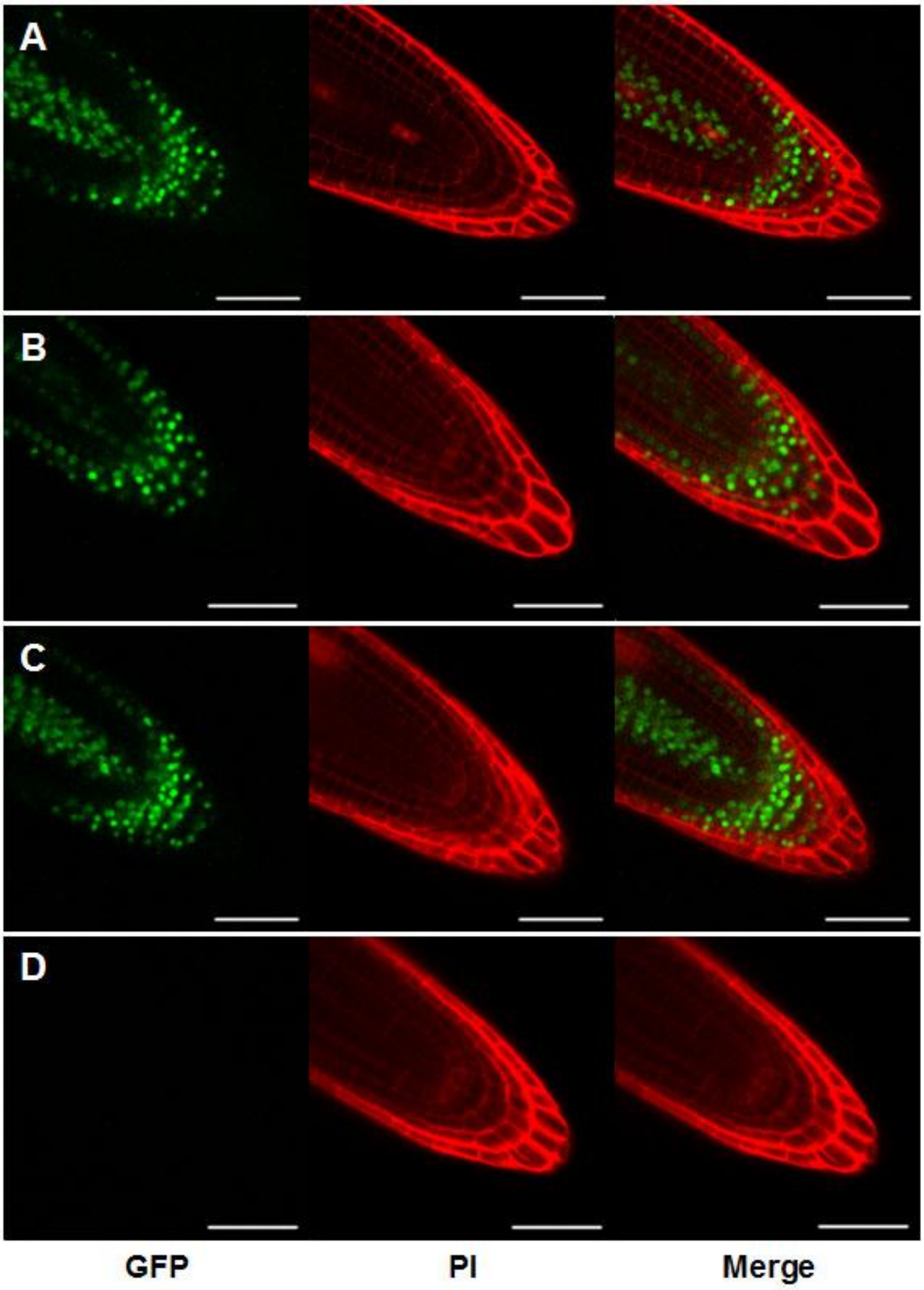

Figure S3. Subcellular localization of *B. napus* TTG2 proteins.

The green fluorescence signal indicates that Bna.TTG2 proteins are located in the nuclei. (A) *BnaA.TTG2.b.1*, (B) *BnaC.TTG2.a.1*, (C) *BnaC.TTG2.b.1*, (D) WT. The GFP image indicating the nuclear localization of Bna.TTG2 proteins are shown on the left column, the propidium iodide images are in the middle, and the merged images are on the right. The bars represent 50 μm.

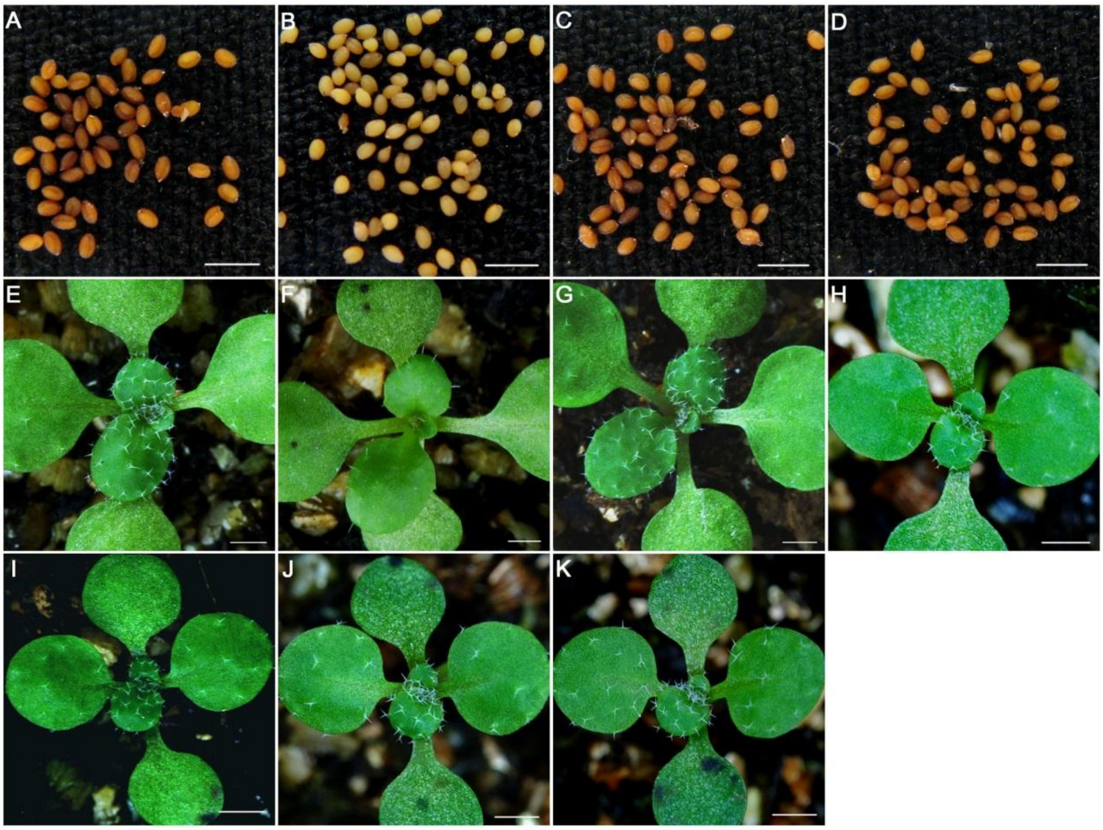

Figure S4. Complementation of the Arabidopsis *ttg2* mutant by *B. napus TTG2* genes.

(A-D) Seed coat pigmentation of WT Arabidopsis (Ler) (A), *ttg2-1* mutant (B), *ttg2-1* mutant expressing *35S:BnaA.TTG2.a.1:GFP* (C) and *ttg2-1* mutant expressing Pro_{AtTTG2}*:AtTTG2:GFP* (D). (E-K) Leaf trichomes of 12-day-old WT Arabidopsis (Ler) (E), *ttg2-1* mutant (F), *ttg2-1* mutant expressing *35S:BnaA.TTG2.a:GFP* (G), *ttg2-1* mutant expressing Pro_{AtTTG2}*:AtTTG2:GFP* (H), *ttg2-1* mutant expressing *35S:BnaA.TTG2.b.1:GFP* (I), *ttg2-1* mutant expressing *35S:BnaC.TTG2.a.1:GFP* (J) and *ttg2-1* mutant expressing *35S:BnaC.TTG2.b.1:GFP* (I). The bars equal 1 mm.

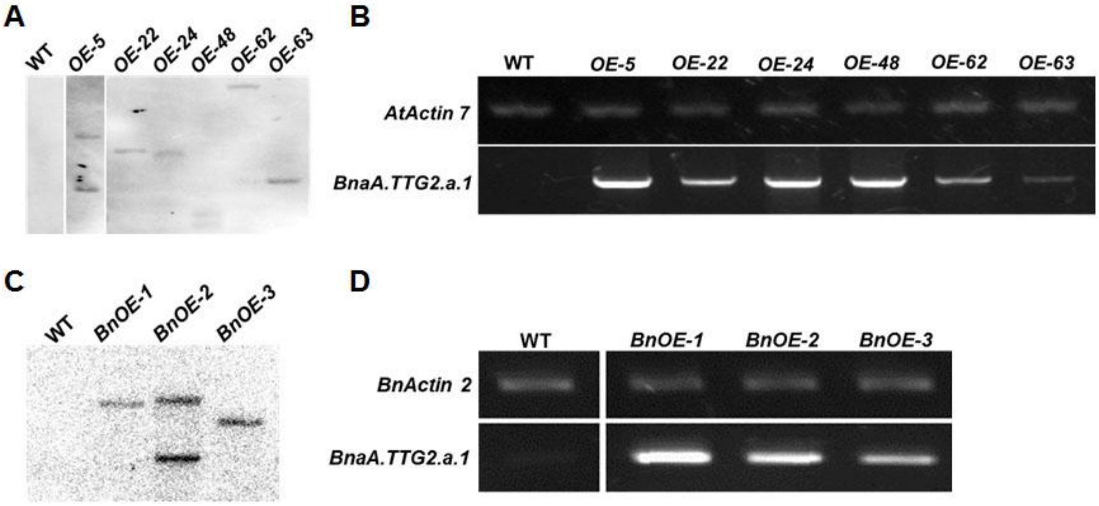

Figure S5. Copy number and expression analysis of *35S:BnaA.TTG2.a.1:GFP* in transgenic Arabidopsis and *B. napus*.

(A) Southern blotting analysis of *BnaA.TTG2.a.1*-overexpressing Arabidopsis lines. (B) RT-PCR analysis of *BnaA.TTG2.a.1*-overexpressing Arabidopsis lines. (C) Southern blotting analysis of *BnaA.TTG2.a.1*-overexpressing *B. napus* lines. (D) RT-PCR analysis of *BnaA.TTG2.a.1*-overexpressing *B. napus* lines.

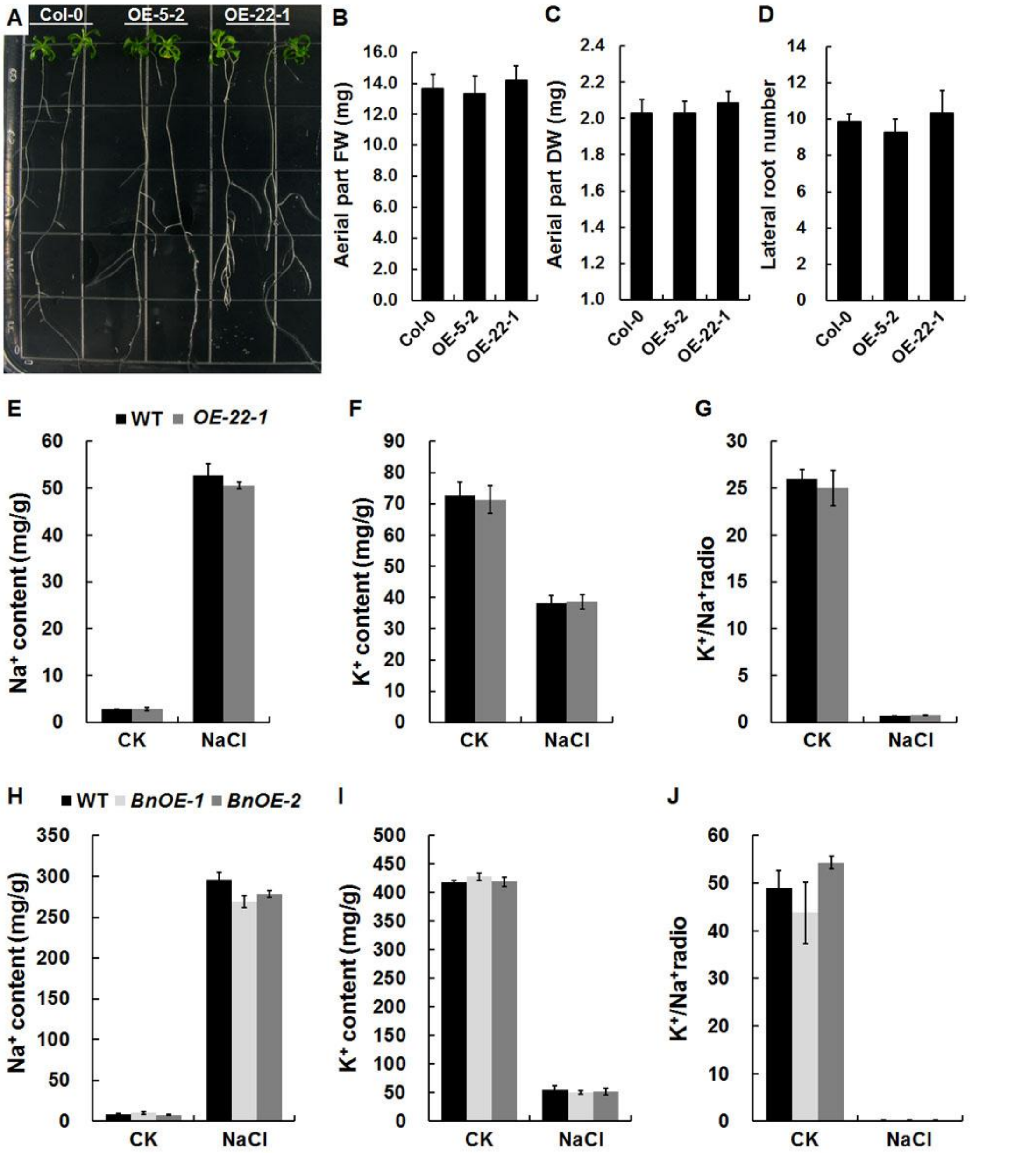

Figure S6. Effects of osmotic stress and ionic toxicity on plant growth in *BnaA.TTG2.a.1*-overexpressing Arabidopsis and *B. napus*.

(A) Four-day-old WT and transgenic Arabidopsis grown on germination media (½ MS) were transferred to ½ MS containing 300 mM mannitol and allowed to grow for an additional 15 days. (B, C) Fresh weight (FW) (B) and Dry weight (DW) (C) of the aerial part of WT and transgenic Arabidopsis treated with ½ MS or ½ MS containing 300 mM mannitol for 15 days (n = 16). (D) Lateral root number of WT and transgenic Arabidopsis in the presence of ½ MS or ½ MS containing 300 mM mannitol for 10 days (n = 20). (E-G) Na^+ content (E), K^+ content (F) and K^+ to Na^+ ratios (G) in WT and OE-22-1 Arabidopsis. (H-J) Na^+

content (H), K^+ content (I) and K^+ to Na^+ ratios (J) in WT and *BnaA.TTG2.a.1*-overexpressing *B. napus*. Data in (B-J) are presented as the means ± SE from three biologically independent experiments.

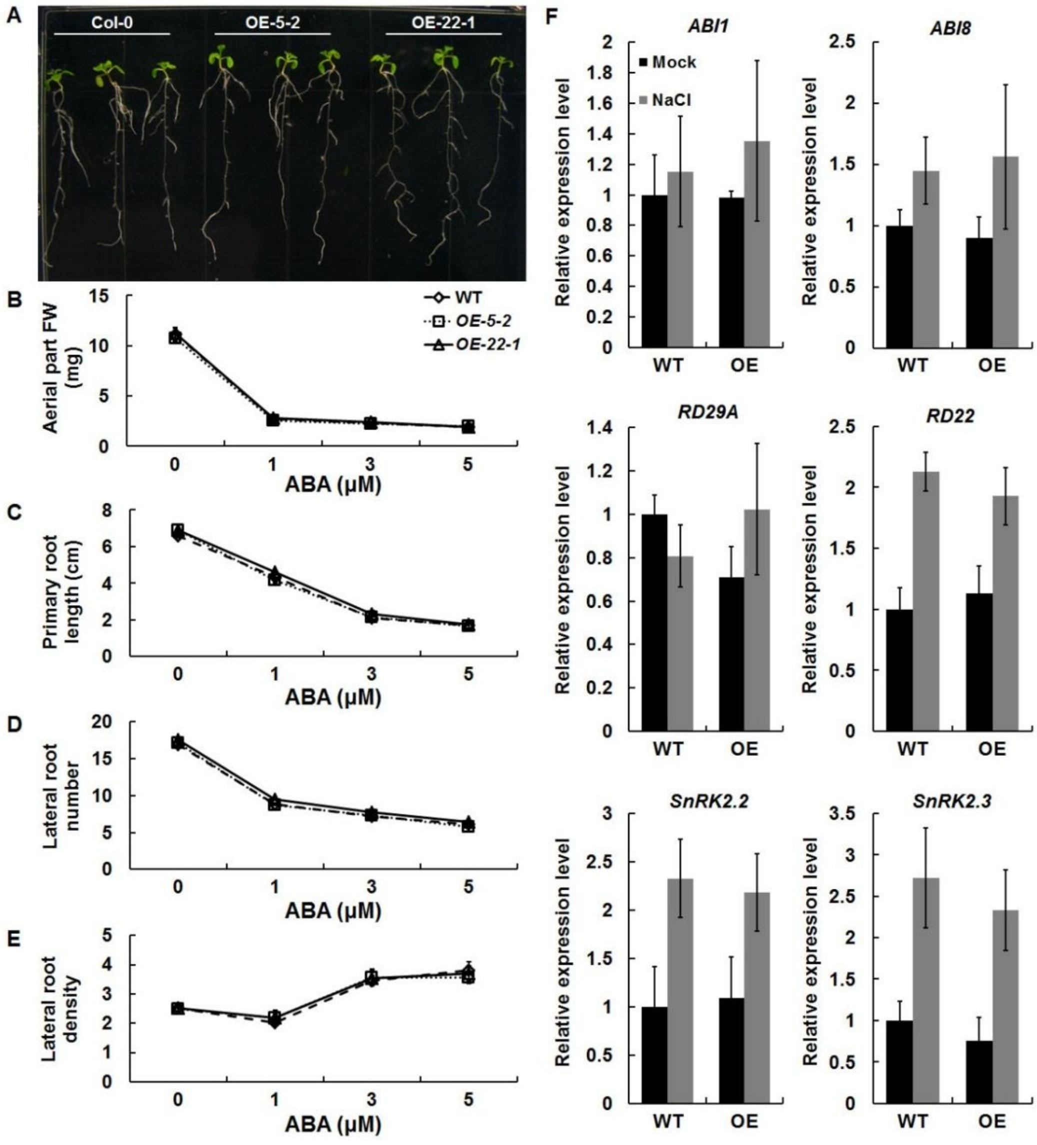

Figure S7. Effects of ABA on plant growth in *BnaA.TTG2.a.1*-overexpressing Arabidopsis.

(A) Four-day-old WT and transgenic Arabidopsis grown on ½ MS were transferred to ½ MS containing 1μM and allowed to grow for an additional 10 days. (B-E) Fresh weight (FW) of the aerial part (B), primary root length (C), lateral root number (D) and lateral root density (E) of the WT and OE plants in ½ MS grown in the presence of ABA. Four-day-old WT and OE plants grown on ½ MS were transferred to ½ MS supplemented with ABA (0, 1, 3 or 5 μM) for another 10 days, the lateral root density was measured on the 7th day, and the aerial part FW was measured on the 10th day (n ≥ 16). (F) Relative expression of ABA response genes in WT and OE plants treated with or

without NaCl. Data in (B-F) are the means ± SE from three biologically independent experiments.

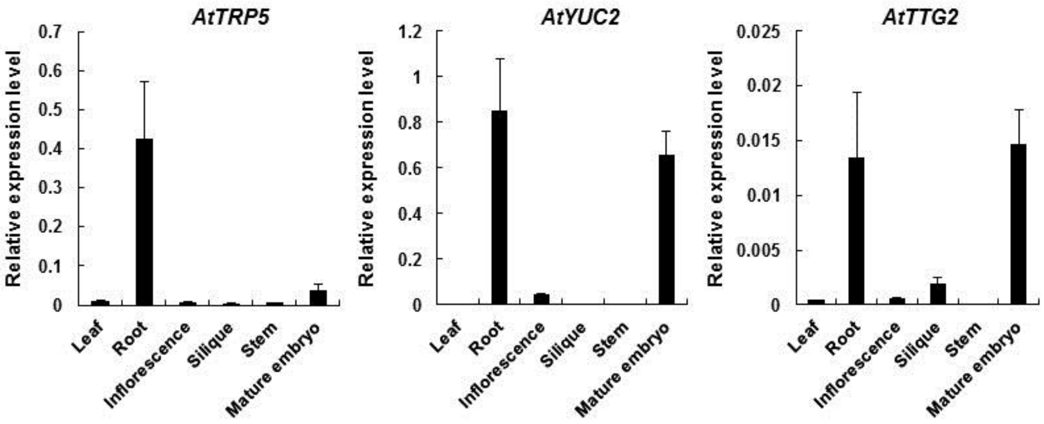

Figure S8. Relative expression of *TRP5* and *YUC2* genes and *AtTTG2* genes in different tissues in WT Arabidopsis.

qRT-PCR analysis of relative expression of *TRP5*, *YUC2* and *AtTTG2* genes in different tissues in WT Arabidopsis. *AtACT7* was used as a normalization control.

Supplementary Methods

Genomic DNA isolation and Southern analysis

Genomic DNA was isolated from the young leaves of *BnaA.TTG2.a.1*-overexpression Arabidopsis, *B. napus* (cultivar J6104), *B. rapa* (accession 1151) and *B. oleracea* (accession T9) by the cetyltrimethylammonium bromide methods (Murray and Thompson, 1980). For the DNA blotting analysis, 20 μg of *B. napus* genomic DNA or 10 μg of Arabidopsis genomic DNA were digested with *Pst*I or *Eco*RI (Fermentas), respectively, and separated on 0.8% agarose gel. After electrophoresis, the digested DNA was transferred onto a Hybond N^+ nylon membrane (Amersham). For hybridization, a 315 bp 3'-terminus conserved *Bna.TTG2* sequence labeled with ^{32}P was used as a probe. The membrane was hybridized for 24 h at 55℃, and then washed twice with 0.1×saline-sodium citrate (SSC) buffer containing 0.1% sodium dodecyl sulfate (SDS) at 65°C for 20 min. The hybridized membrane was scanned with a FUJI FLA-9000 image analysis system (Fujifilm). For Arabidopsis, blotting was performed using a DIG-High Prime DNA Labeling and Detection Starter Kit II (Roche) according to the manufacturer's instructions and using the HPT fragment as a probe. The hybridized membrane was scanned with a FUJI FLA-4000 image analysis system (Fujifilm).

Ion content determination

Sample preparation and ion extraction were essentially performed as described (Nublat *et al.*, 2001). Briefly, NaCl-treated Arabidopsis plants grown in the medium plates or *Brassica* plants grown in hydroponic media (see above) were harvested and washed with ultrapure water for 3 times, and dried at 80°C for 72 h. The dried samples (10-20 mg) were incubated in 0.1 N sulfuric acid at 50°C for 20 min for ion extraction. A flame photometry (Shanghai Precision, FP640) was used for quantitation of Na^+ and K^+ concentrations with NaCl and KCl (Sigma-Aldrich) as the standards.

Construction of plasmids

GUS constructs

Promoter fragments of *BnaA.TTG2.a*, *BnaA.TTG2.b*, *BnaC.TTG2.a* and *BnaC.TTG2.b* were amplified by PCR with the primers LQP43 and LQP50, LQP133 and LQP134, LQP139 and LQP140, LQP137 and LQP138, respectively, using genomic DNA from *B. napus* (cultivar Jia 6104) as the template. The fragments were cloned into a TA cloning vector pMD18-T (Takara) and sequenced. The confirmed promoter fragments were then digested with *Hind*III and *BamH*I and subcloned into the *Hind*III and *BamH*I sites of pBI121 before the *GUS* gene.

Constructs for plant transformation

Full-length *BnaA.TTG2.a.1*, *BnaA.TTG2.b.1*, *BnaC.TTG2.a.1*, *BnaC.TTG2.b.1* were amplified with the primers LQP-AaF and LQP-BT2R, LQP-AbF and LQP-BT2R, LQP-CaF and LQP-BT2R, LQP-CbF and LQP-BT2R, respectively, using cDNA from *B. napus* (cultivar Jia 6104) leaves as template. *BnaA.TTG2.a.1⊿N* was amplified with the primers LQP69 and LQP-BT2R using a *pMD18-T-BnaA.TTG2.a.1* clone as the template. The fragments were cloned to pMD18-T (Takara) and sequenced. The confirmed fragments were then digested with *Pac*I and *Asc*I and subcloned into the corresponding sites of pMDC83 to make a translational fusion to *GFP* (*35S:Bna.TTG2:GFP*). The promoter of *AtTTG2* was amplified with the primers LQP78 and LQP77 using Arabidopsis Col-0 WT genomic DNA as the template. The full-length CDS of *AtTTG2* was amplified with the primers LQP71 and LQP70 using Arabidopsis Col-0 WT cDNA as the template. The fragments were cloned to pMD18-T (Takara) and sequenced. The CDS of *AtTTG2* was digested with *Pac*I and *Asc*I and subcloned into the corresponding sites of pMDC83 vector to make construct pMDC83-*35S:AtTTG2:GFP*. The promoter of *AtTTG2* was digested with *Pme*I and *Pac*I and subcloned into the corresponding sites of pMDC83-*35S:AtTTG2:GFP* vector to make construct pMDC83-$_{Pro}$*AtTTG2:AtTTG2:GFP*.

Constructs for yeast one-hybrid assay

Promoters of Arabidopsis *TRP5* and *YUC2* were amplified with the primers LQP465 and LQP466, LQP461 and LQP462, respectively, using Arabidopsis Col-0 WT genomic DNA as the template. The fragments were cloned to pMD18-T (Takara) and sequenced. The fragments were excised with *Hind*III and *Sal*I and inserted into the corresponding sites of pAbAi (clontech) to form pAbAi-*TRP5* and pAbAi-*YUC2*, respectively. The nucleotide sequences of W-box element 5'-CGTTGACCTTGACCTTGACTTCGTTGACCTTGACCTTGACTT-3' and a mutant W-box element 5'-CGTTGAACTTGAACTTGAATTCGTTGAACTTGAACTTGAATT-3' were synthesized with overhanging sticky ends *Hind*III and *Sal*I. These two elements were inserted into the *Hind*III and *Sal*I sites of pAbAi vector, respectively, to form pAbAi-W-box and pAbAi-mW-box. cDNA fragment *BnaA.TTG2.a.1* was amplified with the primers LQP471 and LQP472 using a cloned BnaA.TTG2.a.1 (pMDC83-*BnaA.TTG2.a.1*) as template and digested with *Nde*I and *BamH*I and subcloned into the corresponding sites of pGADT7 (clontech) vector to form pGADT7-*BnaA.TTG2.a.1*.

Constructs for DLR assay

To make reporter plasmids, promoters of Arabidopsis *TRP5* and *YUC2* were digested from pAbAi-*TRP5* and pAbAi-*YUC2* constructs with *Hind*III and *Sal*I, respectively, and inserted into the *Hind*III and *Sal*I sites of GAL4-LUC vector.

To make effector plasmids, *BnaA.TTG2.a.1:SRDX* fragment was amplified with the primers LQP553 and LQP554 (containing a SRDX (LDLDLELRLGFA) sequence(Hiratsu *et al.*, 2003)) using a cloned BnaA.TTG2.a.1 (pMD18-T-*BnaA.TTG2.a.1*) as the template and digested with *Sac*I and *Sal*I and subcloned into the corresponding sites of pGAL4BD vector (Hao *et al.*, 2010) to substitute the BD domain of the vector to yield 35S:BnaA.TTG2.a.1:SRDX. *BnaA.TTG2.a.1*, *BnaA.TTG2.a.1⊿C1*, *BnaA.TTG2.a.1⊿C2*, *BnaA.TTG2.a.1⊿C3* and *BnaA.TTG2.a.1⊿C4* were amplified with the primer LQP477 and LQP478, LQP477 and LQP499,

LQP477 and LQP500, LQP477 and LQP501, LQP477 and LQP503, respectively, using a cloned *BnaA.TTG2.a.1* (pMD18-T-*BnaA.TTG2.a.1*) as the template and digested with *BamH*I and *Sal*I and subcloned into the corresponding sites of pGAL4BD vector to yield *35S:Gal4BD:Bna.TTG2s*.

Supplement references:

Hiratsu K, Matsui K, Koyama T, Ohme-Takagi M. 2003. Dominant repression of target genes by chimeric repressors that include the EAR motif, a repression domain, in Arabidopsis. *The Plant Journal* **34**, 733-739.

Johnson CS, Kolevski B, Smyth DR. 2002. TRANSPARENT TESTA GLABRA2, a trichome and seed coat development gene of Arabidopsis, encodes a WRKY transcription factor. *The Plant Cell* **14**, 1359-1375.

Murray M, Thompson WF. 1980. Rapid isolation of high molecular weight plant DNA. *Nucleic Acids Research* **8**, 4321-4326.

Nublat A, Desplans J, Casse F, Berthomieu P. 2001. sas1, an Arabidopsis mutant overaccumulating sodium in the shoot, shows deficiency in the control of the root radial transport of sodium. *The Plant Cell* **13**, 125-137.

Supplemental Table S1 List of primers Used in Constructs and Assays

primer	seguence (5' to 3')	Underlined	Destination
Gene cloning			
LQP87	ATGGCTTCTATCTTGAGGTATTCTT		BnaA.TTG2.a F
LQP88	ACGAGCCACAACCATAAACC		BnaA.TTG2.a R
LQP89	ACATCAACGAGCCACAACCC		BnaA.TTG2.b F
LQP90	ATGGCTTGATTAGAATGTTGTGAG		BnaA.TTG2.b R
LQP91	CTTTATCACAAGTGTATGGGTGTCAT		BnaC.TTG2.a F
LQP92	CAATGGGCAATATGGCTTCTATC		BnaC.TTG2.a R
LQP93	AGGGATCTTCCTTTGTCCTATAG		BnaC.TTG2.b F
LQP94	GCTAGGGATTGGTGTTTCTCA		BnaC.TTG2.b R
GUS constructs			
LQP43	AAGCTTACCGCCATTGTTGGTTGCTACTGCT	*Hind* III	*Pro* $_{BnaA.TTG2.a.1}$ *:GUS*
LQP50	GGATCCTATTCTCAATAAAAAAAGTAATCAACCTGA	*BamH* I	*Pro* $_{BnaA.TTG2.a.1}$ *:GUS*
LQP133	CGAAGCTTCTCAAATGGCTAAGCCGGACA	*Hind* III	*Pro* $_{BnaA.TTG2.b.1}$ *:GUS*
LQP134	ATGGATCCAGTCAACCTTAAACAAAAAGATTAAGTTTG	*BamH* I	*Pro* $_{BnaA.TTG2.b.1}$ *:GUS*
LQP139	CGAAGCTTAACAACAATAAAGCCAAATGACTGC	*Hind* III	*Pro* $_{BnaC.TTG2.a.1}$ *:GUS*
LQP140	ATGGATCCTCCCAAAAATAAGTAATTAACCTGAAA	*BamH* I	*Pro* $_{BnaC.TTG2.a.1}$ *:GUS*
LQP137	CGAAGCTTACATACAAACTGAACTCAATCACTGG	*Hind* III	*Pro* $_{BnaC.TTG2.b.1}$ *:GUS*
LQP138	ATGGATCCTATAGTCGTCAACCTTAAACAAATTG	*BamH* I	*Pro* $_{BnaC.TTG2.b.1}$ *:GUS*
Constructs for plant transformation			
LQP-AaF	TTAATTAAATGGATGTGAAAGAGAGTGAAA	*Pac* I	*35S:BnaA.TTG2.a.1:GFP*
LQP-AbF	TTAATTAAATGGAGGTGAAAGAGAGTAAGA	*Pac* I	*35S:BnaA.TTG2.b.1:GFP*
LQP-CaF	TTAATTAAATGGATGTGAAAGAGAGGGAAA	*Pac* I	*35S:BnaC.TTG2.a.1:GFP*
LQP-CbF	TTAATTAAATGGAGGTGAAAGAGAGTAAAA	*Pac* I	*35S:BnaC.TTG2.b.1:GFP*
LQP-BT2R	GGCGCGCCAAATGGCTTGATTAGAATGTTGTG	*Asc* I	*35S:Bna.TTG2s:GFP*
LQP69	TTAATTAAATGTCTTGTGATGATGATTCAGATAGC	*Pac* I	*35S:BnaA.TTG2.a.1 △ N:GFP*

LQP78	GTTTAAACTCACCTCAATAGAAATAATCATACACACTT	*Pme* I	Pro_{AtTTG2} *:GUS*
LQP77	TTAATTAATACTTGACCTTAATCAATTATGTAAGAGAC	*Pac* I	Pro_{AtTTG2} *:GUS*
LQP71	TTAATTAAATGGAGGTGAATGATGGTGAAAGAG	*Pac* I	Pro_{AtTTG2} *:AtTTG2:GFP*
LQP70	GGCGCGCCAAATTGTTTGCTTAGAAAGTTGTGG	*Asc* I	Pro_{AtTTG2} *:AtTTG2:GFP*
Constructs for yeast one-hybrid assay			
LQP465	GCAAGCTT ACCGCACAATAAGAAATCAGAGA	*Hind* III	pAbAi-*TRP5*
LQP466	TAGTCGACTGTAACGGCTAAGAACTCGGTG	*Sal* I	pAbAi-*TRP5*
LQP461	GCAAGCTTTGGAAAATACAGAAGCCAAAAGAA	*Hind* III	pAbAi-*YUC2*
LQP462	TAGTCGACGAAAGAGAGAAAGAGAAGAAAAAAGAGA	*Sal* I	pAbAi-*YUC2*
LQP513	AGCTTCGTTGACCTTGACCTTGACTTCGTTGACCTTGACCTTGACTTG	*Hind* III sticky end	pAbAi-*W-box*
LQP514	TCGACAAGTCAAGGTCAAGGTCAACGAAGTCAAGGTCAAGGTCAACGA	*Sal* I sticky end	pAbAi-*W-box*
LQP515	AGCTTCGTTGAACTTGAACTTGAATTCGTTGAACTTGAACTTGAATTG	*Hind* III sticky end	pAbAi-*mW-box*
LQP516	TCGACAATTCAAGTTCAAGTTCAACGAATTCAAGTTCAAGTTCAACGA	*Sal* I sticky end	pAbAi-*mW-box*
LQP471	TACATATGGATGTGAAAGAGAGTGAAAGAA	*Nde* I	pGADT7-*BnaA.TTG2.a.1*
LQP472	TAGGATCCTTAAATGGCTTGATTAGAATGTTGTG	*BamH* I	pGADT7-*BnaA.TTG2.a.1*
Constructs for electrophoretic mobility shift assays			
LQP737	TTCAAGAAAATGTCAAAGTTTCTACATTCGTACTATTATATAATTGACTG AAGAAATTA		*TRP5* F for EMSA
LQP738	TAATTTCTTCAGTCAATTATATAATAGTACGAATGTAGAAACTTTGACATT TTCTTGAA		*TRP5* R for EMSA
LQP741	TTCAAGAAAAGGGGGGAGTTTCTACATTCGTACTATTATATAAGGGGGGG AAGAAATTA		*TRP5* -m F for EMSA
LQP742	TAATTTCTTCCCCCCCTTATATAATAGTACGAATGTAGAAACTCCCCCCTT TTCTTGAA		*TRP5-m* R for EMSA
LQP723	TGGTAATCATAGTACACCAAGTTGACTGCACAGTGATAACATATATAT		*YUC2* F for EMSA
LQP724	ATATATATGTTATCACTGTGCAGTCAACTTGGTGTACTATGATTACCA		Y*UC2* R for EMSA
LQP751	TGGTAATCATAGTACACCAAGGGGGGGGCACAGTGATAACATATATAT		*YUC2* -m F for EMSA
LQP752	ATATATATGTTATCACTGTGCCCCCCCCTTGGTGTACTATGATTACCA		*YUC2* -m R for EMSA
LQP733	CGTTGACCTTGACCTTGACTTCGTTGACCTTGACCTTGACTT		*W-box* F for EMSA

LQP734	AAGTCAAGGTCAAGGTCAACGAAGTCAAGGTCAAGGTCAACG		*W-box R for* EMSA
LQP735	CGTTGAACTTGAACTTGAATTCGTTGAACTTGAACTTGAATT		*mW-box* F for EMSA
LQP736	AATTCAAGTTCAAGTTCAACGAATTCAAGTTCAAGTTCAACG		*mW-box* R for EMSA
Constructs for DLR assay			
LQP553	TAGAGCTCATGGATGTGAAAGAGAG	*Sac* I	*35S:BnaA.TTG2.a.1:SRDX*
LQP554	TAGTCGACTTACGCAAAGCCCAGGCGCAGTTCCAGATCCAGATCCAGAAT GGCTTGATTAGAATGTT	*Sal* I; SRDX sequence	*35S:BnaA.TTG2.a.1:SRDX*
LQP477	TAGAGCTCATGGATGTGAAAGAGAGTGAAAGAA	*BamH* I	*35S:Gal4BD:Bna.TTG2s*
LQP478	TAGTCGACTTAAATGGCTTGATTAGAATGTTGTG	*Sal* I	*35S:Gal4BD:BnaA.TTG2.a.1*
LQP499	TAGTCGACTTAGAGACTGTCTGATTCCCCTGAG	*Sal* I	*35S:Gal4BD:BnaA.TTG2.a.1 △C1*
LQP500	TAGTCGACTTAACAAGAGGGTTTAGAGTGATTA	*Sal* I	*35S:Gal4BD:BnaA.TTG2.a.1 △C2*
LQP501	TAGTCGACTTATTGGGAGACAAGCTTTGCTTTA	*Sal* I	*35S:Gal4BD:BnaA.TTG2.a.1 △C3*
LQP503	TAGTCGACTTATTGTGGAGAGACAGTAACTGAA	*Sal* I	*35S:Gal4BD:BnaA.TTG2.a.1 △C4*
Primers for RT-PCR			
LQP21	ATGGATGTGAAAGAGAGTGAAA		RT-PCR for BnaA.TTG2.a.1 OE plants
LQP22	TTAAATGGCTTGATTAGAATGTTGTG		RT-PCR for BnaA.TTG2.a.1 OE plants
Primers for real-time PCR			
LQP309	CTAACAAACGGCGTGAGTGG		*TRP5*
LQP310	CTCTGTTCTTGGGCACAATCAT		*TRP5*
LQP317	AGCCATTGATGCCAAGAAGA		*YUC2*
LQP318	AATCCAAACTTGCCAAATCG		*YUC2*
LQP419	ATCCGACGCTCCTGCTCTA		*IAA2*
LQP420	ACTCCGATCCGAATCAAACC		*IAA2*
LQP259	AAAGGTTTAGGCTGTGGTGTATGAG		*IAA3*
LQP260	CCGATGAGGTAAATAAAGGCTATGA		*IAA3*
LQP423	AGGTTTAGGTTGTGGTGGTCTTT		*IAA4*
LQP424	TCTCTCCCCAAGAACCCGA		*IAA4*
LQP273	GAAGGTATCAATGGACGGAGCAC		*IAA17*
LQP274	CCGACGAGCATCCAATCACC		*IAA17*

LQP263	ACTGGATTATTAGATGGGAATGGAG	*IAA18*
LQP264	TCTTCTCATTTTCTCTTGCTTACCA	*IAA18*
LQP351	GTCAAGAGCAGAAGGATAGACAAAG	*IAA12*
LQP352	GAAACAAACTGGAAAACTACGAATT	*IAA12*
LQP627	CTTGAACTGAGGCAGAGAGGGT	*ABI1*
LQP628	AAGGGGCTTTTTAGAATGTAGTCAA	*ABI1*
LQP721	CAGCCATCCCTCCTTTATCG	*ABI8*
LQP722	GCCGCTTTCTTCACTCTTCTTT	*ABI8*
LQP239	GTGCGACGGAGGAGGTGAA	*RD29A*
LQP240	TCCAAAGCCGAACAATTTATTAAC	*RD29A*
LQP663	TAAAGGTGAAGCCAGGGACC	*RD22*
LQP664	TCTTATATGGAAACCACGCATTACTA	*RD22*
LQP649	GAGATTGATGTTGACAGTAGTGGAGA	*SnRK2.2*
LQP650	ACAACAAACGATCCAAAAATGAA	*SnRK2.2*
LQP651	ACATTGACAGTAGCGGAGAGATAGT	*SnRK2.3*
LQP652	AGAAAACAAGAAACAAACAGAGACAA	*SnRK2.3*
LQP637	ACAACCTACGAGGGTAAACATAATCA	*AtTTG2*
LQP638	CTTAGGAAGTTGTGAGTGAAGAGCA	*AtTTG2*
Primers for probe synthesis		
HPTF	GATGTTGGCGACCTCGTATT	probe synthesis of *HPT* gene
HPTR	GTGTCACGTTGCAAGACCTG	probe synthesis of *HPT* gene
LQP65	AAGAACGAGAAGCAATCAAG	probe synthesis of *Bna.TTG2s*
LQP46	TTAAATGGCTTGATTAGAATGTTGTG	probe synthesis of *Bna.TTG2s*

Supplemental Table S2 Measurements of endogenous IAA contents in WT and OE plants under the conditions with or without salt stress.

Biological replication	Sample	Calculated Concentration (ng/mL)[1]	Weight (g)[2]	IAA content (ng/g)[3]	Mean ± SE (ng/g)[4]	P value[5]
**Plants grown on ½ MS**						
1	WT-1	3.89	0.0695	16.79136691		
2	WT-2	4.39	0.0726	18.14049587		
3	WT-3	3.77	0.0675	16.75555556		
4	WT-4	4.28	0.0714	17.98319328		
5	WT-5	3.1	0.0619	15.02423263	16.93896885±0.559272446	
1	OE-5-1	2.69	0.0444	18.17567568		
2	OE-5-2	3.33	0.0662	15.09063444		
3	OE-5-3	3.02	0.0556	16.29496403		
4	OE-5-4	3.03	0.0637	14.2700157		
5	OE-5-5	2.99	0.0523	17.15105163	16.19646829±0.699009325	0.43092
1	OE-22-1	3.17	0.0601	15.82362729		
2	OE-22-2	2.99	0.0511	17.55381605		
3	OE-22-3	3.72	0.0626	17.82747604		
4	OE-22-4	3.13	0.0647	14.51313756		
5	OE-22-5	2.2	0.0405	16.2962963	16.40287065±0.602998671	0.532777
**Plants grown on ½ MS+75mM NaCl**						
1	NaCl-WT-1	3.99	0.0757	15.81241744		
2	NaCl-WT-2	3.2	0.061	15.73770492		
3	NaCl-WT-3	3.77	0.063	17.95238095		
4	NaCl-WT-4	4.44	0.0785	16.96815287	16.61766404±0.526550235	
1	NaCl-OE-5-1	3.02	0.0679	13.34315169		
2	NaCl-OE-5-2	2.65	0.0596	13.33892617		
3	NaCl-OE-5-3	2.35	0.0604	11.67218543		
4	NaCl-OE-5-4	2.8	0.0711	11.81434599	12.54215232±0.462150139	0.001134

1	NaCl-OE-22-1	3.16	0.0882	10.74829932			
2	NaCl-OE-22-2	2.78	0.0604	13.80794702			
3	NaCl-OE-22-3	3.62	0.0811	13.39087546			
4	NaCl-OE-22-4	2.74	0.0608	13.51973684	12.86671466	±0.71149984	0.005454
**IAA standard for standard curve**							
	standard-0.025 ppb	0.0255					
	standard-0.05 ppb	0.0518					
	standard-0.1 ppb	0.124					
	standard-0.2 ppb	0.181					
	standard-0.5 ppb	0.492					
	standard-1 ppb	0.998					
	standard-3 ppb	3					

[1]Sample concentrations calculated based on the external standard curve with a D2-IAA internal standard correction with the quantification software included in the mass spectrometer used for the assay.

[2]Fresh weights of Samples prepared as described in Materials and Methods.

[3]IAA content per sample calculated using the following formulas: calculated concentration × 0.3 ml (volume of resuspend buffer when extracting, see materials and methods) /

[4]Average and standard error calculated from indicated biological replications.

[5]P value for two-tailed unpaired Student’s t-tests. The comparison was made between WT and each OE line in two growth conditions (with and without NaCl), respectively.
